# An adaptive model predictive control approach for robust load frequency control under renewable energy disturbances

**DOI:** 10.1038/s41598-025-33986-5

**Published:** 2026-01-16

**Authors:** Mohamed Ayman, Mahmoud A. Attia, Ahmed M. Asim

**Affiliations:** Department of Electrical Power and Machines, Faculty of Engineering, Ain Shams University, Cairo, Egypt

**Keywords:** Energy science and technology, Engineering, Mathematics and computing

## Abstract

This paper presents an Adaptive Model Predictive Control (AMPC) strategy for robust load–frequency control (LFC) in single-area and double-area power systems under load variations, parameter uncertainty, and renewable energy disturbances. The controller integrates online system identification using Recursive Least Squares (RLS) with a receding-horizon optimization framework to ensure real-time model adaptation and constraint-aware predictive regulation. Simulation results demonstrate that the proposed AMPC significantly improves transient and steady-state performance compared with conventional PI/PID controllers. In single-area systems, the AMPC achieves settling times of 0.5–1 s, compared with 30 s for PI, and eliminates overshoot while reducing undershoot from 4.5 × 10⁻³ to 1 × 10⁻³. Under dynamic and wind disturbances, peak-to-peak deviations are reduced to **≈ 0**, whereas PI exhibits deviations up to 26.5 × 10⁻³. In double-area systems, the AMPC reduces settling time from 20 to 40 s (PID) to 1–2 s and minimizes undershoot by up to an order of magnitude. Comparative studies further confirm the proposed AMPC’s superiority over Harmony Search (HS), Sine–Cosine Algorithm (SCA), Teaching–Learning-Based Optimization (TLBO)-optimized PID/PIDA controllers and the Marine Predator Algorithm (MPA)-based cascaded PIDA, establishing AMPC as an effective and scalable solution for low-inertia grids with high renewable penetration.

## Introduction

 The modern electrical power system is one of the most complex and critical infrastructures ever developed, serving as the backbone of industrial, commercial, and residential activities worldwide. Its primary objective is to ensure a continuous and high-quality supply of electrical energy to consumers. A fundamental indicator of this quality is the stability of the system’s operating frequency. In any interconnected Alternating Current (AC) power grid, frequency serves as a global indicator of the real-time balance between active power generation and consumption. Maintaining this frequency close to its nominal value is essential, as significant deviations can degrade the performance of sensitive equipment, trigger protective relays leading to load shedding, or even cause cascading failures that result in widespread blackouts^1,2^. The core mechanism responsible for maintaining this balance is the Load Frequency Control (LFC) system. LFC has two main objectives: first, to minimize transient frequency deviations and restore nominal frequency in a stable and timely manner after load or generation disturbances; and second, in interconnected systems, to maintain scheduled power exchanges between control areas. Each area continuously calculates its Area Control Error (ACE), a signal combining frequency deviation and tie-line power deviation. The LFC system then issues corrective control signals to participating generators to drive the ACE toward zero, thereby restoring both frequency and power exchange to their scheduled values^2–4^. Traditionally, power grids were dominated by large synchronous generators (thermal and hydro), whose substantial rotational inertia naturally buffered frequency fluctuations. However, the global transition toward de-carbonization has led to the large-scale integration of Renewable Energy Sources (RES) such as wind and solar photovoltaics. These sources, typically interfaced through power electronic converters, lack inherent inertia, thereby reducing overall system stability and making grids more susceptible to frequency excursions. Moreover, the stochastic and intermittent behavior of RES introduces additional uncertainty, compounded by emerging dynamic load patterns—such as those caused by electric vehicle charging—and the complexities of deregulated electricity markets^5,6^. Recent studies have emphasized the importance of considering wind-penetration uncertainty in LFC design^7^. demonstrates the worst-case of wind-penetration modeling significantly enhances robustness and damping performance in multi-area hybrid systems. Conventional Proportional–Integral (PI) and Proportional–Integral–Derivative (PID) controllers have long been the standard approach for LFC due to their simplicity, ease of implementation, and low computational cost. However, as these controllers are designed based on linearized system models around specific operating points, their performance deteriorates under large disturbances or varying conditions due to nonlinearities and parameter uncertainties. In modern low-inertia grids, the fixed-gain nature of these controllers often results in large overshoots, slow settling times, and poor disturbance rejection^8^. Disturbance-observer (DOB) techniques have also been introduced to improve frequency regulation under uncertainties and communication delays^9^. achieves faster disturbance rejection and improved robustness compared to classical PI-based methods. To overcome these challenges, researchers have explored advanced and intelligent control paradigms. Notable approaches include Fuzzy Logic Controllers (FLC) and Artificial Neural Networks (ANN), which handle nonlinearities without precise mathematical modeling, and robust control methods such as Sliding Mode Control (SMC) and H-infinity (H∞) control, which ensure stability across uncertainties. More recently, Fractional-Order PID (FOPID) controllers have gained attention for their ability to fine-tune dynamic responses using fractional calculus, achieving greater robustness and flexibility than traditional controllers^10–14^. The increasing complexity of these advanced controllers makes manual tuning impractical, motivating the integration of metaheuristic optimization algorithms for optimal LFC design. These population-based algorithms efficiently navigate complex, non-convex search spaces to find near-global optimal controller parameters that minimize predefined performance indices, such as time-domain error criteria. Over time, optimization techniques have evolved from classical methods like Genetic Algorithms (GA) and Particle Swarm Optimization (PSO) to more recent and powerful approaches, including the Grey Wolf Optimizer (GWO), Whale Optimization Algorithm (WOA), Ant Lion Optimizer (ALO), and Slap Swarm Algorithm (SSA), which have demonstrated excellent performance in designing high-quality LFC schemes for multi-area, multi-source power systems^15–21^.

### Research gap and contribution

Traditional LFC strategies based on fixed-gain PI or PID controllers are inadequate for modern power systems characterized by high renewable penetration and dynamic operating conditions. These conventional methods fail to adapt to system nonlinearities and time-varying parameters, resulting in degraded frequency response under disturbances or changing operating points. Although several intelligent and robust control techniques have been proposed, most lack real-time adaptability and model-based predictive capability. The present study addresses this gap by developing an Adaptive Model Predictive Controller (AMPC) that continuously updates its internal model using online RLS-based system identification. This adaptation enables precise prediction and control of system dynamics, maintaining frequency stability even under significant load variations and renewable-induced disturbances. Thus, the proposed AMPC bridges the gap between robustness, adaptability, and predictive control in modern interconnected grids.

## Proposed adaptive model predictive controller (AMPC)

### Overview and motivation

In many practical applications, system parameters vary over time due to nonlinearities, load changes, or environmental conditions. A conventional Model Predictive Controller (MPC) assumes a fixed linear model, which can lead to poor control performance when the real plant deviates from this model.

The proposed Adaptive Model Predictive Controller (AMPC) is designed to overcome this limitation by continuously updating the prediction model according to the current operating conditions. This allows the controller to maintain accurate predictions, satisfy system constraints, and achieve robust performance under parameter variations^22,23^.

### Time-varying state-space model

The AMPC framework uses a discrete-time, linear time-varying (LTV) state-space representation of the plant as follows in Eqs. (1)&(2)^24^:

1 $$\:\mathrm{x}\left(\mathrm{k}+1\right)={\mathrm{A}}_{\mathrm{k}}\text{}\mathrm{x}\left(\mathrm{k}\right)+{\mathrm{B}}_{\mathrm{k}}\text{}\mathrm{u}\left(\mathrm{k}\right)+{\mathrm{E}}_{\mathrm{k}}\text{}\mathrm{d}\left(\mathrm{k}\right)\:\text{}$$

2 $$\:\mathrm{y}\left(\mathrm{k}\right)={\mathrm{C}}_{\mathrm{k}}\text{}\mathrm{x}\left(\mathrm{k}\right)$$

where

- $\:x\left(k\right)\:$is the state vector,
- $\:u\left(k\right)\:$is the control input,
- $\:d\left(k\right)\:$represents measurable or unmeasurable disturbances,
- $\:y\left(k\right)\:$is the plant output.
- The matrices $\:{A}_{k}\text{},{B}_{k}\text{},{C}_{k}\text{},{E}_{k}$​ are updated at each sampling instant to reflect the current dynamics of the plant.

The model parameters are identified online using recursive estimation techniques (e.g., Recursive Least Squares or Extended Kalman Filter) to capture time-varying system behavior^25^.

Only the latest model $\:\left({A}_{k}\text{},{B}_{k}\text{},{C}_{k\text{}}\right)\:$is used during the current prediction horizon, following the receding-horizon principle of MPC.

### Online RLS-based system identification

To enable continuous adaptation of the predictive model, the plant parameters are identified online using the Recursive Least Squares (RLS) algorithm. The RLS estimator updates a parameter vector $\:\theta\:\left(k\right)$ that represents the dominant coefficients of the governor–turbine–generator subsystem. The algorithm follows the standard recursive update Eqs. (3),(4),(5)&(6)^24,25^:

3 $$\:\mathrm{e}\left(\mathrm{k}\right)=\mathrm{y}\left(\mathrm{k}\right)-{{\upvarphi\:}}^{\mathrm{T}}\left(\mathrm{k}\right){\uptheta\:}(\mathrm{k}-1)$$

4 $$\:\mathrm{K}\left(\mathrm{k}\right)=\frac{\mathrm{P}\left(\mathrm{k}-1\right){\upvarphi\:}\left(\mathrm{k}\right)}{{\uplambda\:}+{{\upvarphi\:}}^{\mathrm{T}}\:\left(\mathrm{k}\right)\mathrm{P}\left(\mathrm{k}-1\right){\upvarphi\:}\left(\mathrm{k}\right)}\text{}$$

5 $$\:{\uptheta\:}\left(\mathrm{k}\right)={\uptheta\:}(\mathrm{k}-1)+\mathrm{K}\left(\mathrm{k}\right)\mathrm{e}\left(\mathrm{k}\right)$$

6 $$\:\mathrm{P}\left(\mathrm{k}\right)=\frac{1}{{\uplambda\:}}\text{}\left[\mathrm{P}\right(\mathrm{k}-1)-\mathrm{K}\left(\mathrm{k}\right){{\upvarphi\:}}^{\mathrm{T}}(\mathrm{k}\left)\mathrm{P}\right(\mathrm{k}-1\left)\right]$$

The forgetting factor $\:\lambda\:$ typically ($\:0.98\le\:\lambda\:\le\:1$) determines how fast the estimator tracks time-varying parameters. The identified parameter vector is mapped to the time-varying state-space matrices ($\:A\left(k\right),B\left(k\right),C\left(k\right)$), which are updated at each sampling instant to provide the AMPC with an accurate prediction model^25^. Parameter bounding and covariance resetting are applied to enhance robustness under noise and sudden variations^25^.

### Nominal deviation form

For constraint handling, the plant model is written in deviation form with respect to a nominal operating point$\:({x}_{0}\text{},{u}_{0}\text{},{y}_{0}\text{})$ as in Eqs. (7)&(8) :

7 $$\:{\Delta\:}\mathrm{x}(\mathrm{k}+1)={\mathrm{A}}_{\mathrm{k}}\text{}{\Delta\:}\mathrm{x}\left(\mathrm{k}\right)+{\mathrm{B}}_{\mathrm{k}}\text{}{\Delta\:}\mathrm{u}\left(\mathrm{k}\right)$$

8 $$\:{\Delta\:}\mathrm{y}\left(\mathrm{k}\right)={\mathrm{C}}_{\mathrm{k}}\text{}{\Delta\:}\mathrm{x}\left(\mathrm{k}\right)$$

Where

$$\:{\Delta\:}\mathrm{x}\left(\mathrm{k}\right)=\mathrm{x}\left(\mathrm{k}\right)-{\mathrm{x}}_{0}\text{},\:\varDelta\:u\left(k\right)=u\left(k\right)-{u}_{0}\:,\:\mathrm{a}\mathrm{n}\mathrm{d}\:\varDelta\:y\left(k\right)=y\left(k\right)-{y}_{0}.$$.

This formulation simplifies the control optimization problem and ensures that the controller reacts only to deviations from nominal conditions^24^.

### Receding-horizon optimization

At each sampling instant k, the AMPC computes a control sequence that minimizes a finite-horizon cost function as in Eq. (9):

9 $$\:\mathrm{J}=\sum\limits_{\mathrm{i}=1}^{\mathrm{N}\mathrm{p}}\parallel\:\mathrm{y}\left(\mathrm{k}+\mathrm{i}\right)-\mathrm{r}\left(\mathrm{k}+\mathrm{i}\right){\parallel\:}_{\mathrm{Q}}^{2}\text{}\text{}+\sum\limits_{\mathrm{i}=0}^{\mathrm{N}\mathrm{c}-1}\parallel\:{\Delta\:}\mathrm{u}\left(\mathrm{k}+\mathrm{i}\right){\parallel\:}_{\mathrm{R}}^{2}\text{}$$

where $\:{N}_{p}$​ is the prediction horizon, $\:{N}_{c}$​ is the control horizon,

$\:Q$ and $\:R$ are weighting matrices, and $\:r\left(k\right)$ is the reference signal.

The optimization is subject to the following constraints such as:

$$\:{\mathrm{u}}_{min}\:\text{}\le\:\mathrm{u}\left(\mathrm{k}+\mathrm{i}\right)\le\:{\mathrm{u}}_{max}\:\text{},{\Delta\:}{\mathrm{u}}_{min}\text{}\le\:{\Delta\:}\mathrm{u}\left(\mathrm{k}+\mathrm{i}\right)\le\:{\Delta\:}{\mathrm{u}}_{max}\:\text{},\:{\mathrm{y}}_{min}\text{}\le\:\mathrm{y}(\mathrm{k}+\mathrm{i})\le\:{\mathrm{y}}_{max}$$

Only the first control move $\:u\left(k\right)$ is applied, while the optimization problem is solved again at the next time step using the updated plant model (receding-horizon principle)^22,26^.

### State estimation (time-varying kalman filter)

Since all system states are not directly measurable, the AMPC uses a linear time-varying Kalman filter (LTVKF) to estimate them^23^.

At each time step k, the filter updates the state estimate $\:x\left(k\right)$ and the covariance matrix $\:P\left(k\right)$ according to the current plant model.

The key recursive equations are:

1. Prediction step as in Eq. (10)

10 $$\:{\mathrm{P}}_{\mathrm{k}|\mathrm{k}-1}={\mathrm{A}}_{\mathrm{k}}{\mathrm{P}}_{\mathrm{k}-1|\mathrm{k}-1}{\mathrm{A}}_{\mathrm{k}}^{\mathrm{T}}+\mathrm{Q}$$

2. Kalman gains as in Eqs. (11)&(12)

11 $$\:{\mathrm{L}}_{\mathrm{k}}=\:\left({\mathrm{A}}_{\mathrm{k}}{\mathrm{P}}_{\mathrm{k}|\mathrm{k}-1}\:{\mathrm{C}}_{\mathrm{k}}^{\mathrm{T}}+\:\mathrm{N}\right){\left({\mathrm{C}}_{\mathrm{k}}{\mathrm{P}}_{\mathrm{k}|\mathrm{k}-1}\:{\mathrm{C}}_{\mathrm{k}}^{\mathrm{T}}+\:\mathrm{R}\right)}^{-1}\:$$

12 $$\:{\mathrm{M}}_{\mathrm{k}}\:=\:{\mathrm{P}}_{\mathrm{k}|\mathrm{k}-1}\:\:\:{\mathrm{C}}_{\mathrm{k}}^{\mathrm{T}}\:{\left({\mathrm{C}}_{\mathrm{k}}\:{\mathrm{P}}_{\mathrm{k}|\mathrm{k}-1}\:\:{\mathrm{C}}_{\mathrm{k}}^{\mathrm{T}}\:+\:\mathrm{R}\right)}^{-1}\:$$

3. Covariance update as in Eq. (13)

13 $$\:{\mathrm{P}}_{\mathrm{k}+1|\mathrm{k}}\:\:=\:{\mathrm{A}}_{\mathrm{k}}\:{\mathrm{P}}_{\mathrm{k}|\mathrm{k}-1}\:{\mathrm{A}}_{\mathrm{k}}^{\mathrm{T}}\:-\:{\left({\mathrm{A}}_{\mathrm{k}}\:{\mathrm{P}}_{\mathrm{k}|\mathrm{k}-1}\:{\mathrm{C}}_{\mathrm{k}}^{\mathrm{T}}\:+\:\mathrm{N}\right)\mathrm{L}}_{\mathrm{k}}^{\mathrm{T}}\:+\:\mathrm{Q}$$

Here, $\:Q,R$ and $\:N$ are covariance matrices representing process noise, measurement noise, and cross-correlation, respectively.

When the model remains constant, the LTVKF converges to the traditional steady-state Kalman filter used in standard MPC^23,25^.

### Stability and robustness

The AMPC maintains recursive feasibility and closed-loop stability by updating model parameters gradually and ensuring constraint satisfaction at all times.

Robustness is achieved by:

- Applying tightened constraint sets around the nominal trajectory^24^.
- Monitoring model parameter changes and triggering conservative fallback control if large deviations occur^25^.
- Including terminal costs and stability constraints in the optimization problem^26^.

This ensures stable operation even under significant system variations and modeling uncertainty.

### Implementation steps of the adaptive control strategy

The complete implementation of the proposed AMPC strategy follows the steps below:

1. **Initialization**:
  The initial state-space model $\:({A}_{0},\:{B}_{0},\:{C}_{0})$, RLS parameters $\:{\theta\:}_{0}$​, forgetting factor $\:\lambda\:$, and the MPC horizons ($\:{N}_{p}$,$\:{N}_{c}$) are initialized at $\:k=0$.

2. **State Measurement**:
  At each sampling instant, the measurable outputs (frequency deviation and tie-line power deviation) are fed back to the controller.

3. **Online Identification (RLS)**:
  The RLS estimator updates the parameter vector $\:\theta\:\left(k\right)$ and regenerates the time-varying state-space matrices $\:(A\left(k\right),B\left(k\right),C\left(k\right))$.

4. **State Estimation**:
  The updated model is used by the time-varying Kalman filter to obtain the estimated states $\:x\left(k\right)$.

5. **MPC Optimization**:
  Using the updated model, the AMPC solves the constrained finite-horizon optimization problem and computes the optimal control sequence.

6. **Control Application**:
  Only the first element of the optimal sequence is applied to the plant (“receding horizon”), ensuring closed-loop operation.

7. **Model Update**:
  Steps 2–6 repeat at every sampling instant, allowing the controller to adapt to parameter variations, disturbances, and nonlinear operating conditions.

This implementation ensures real-time adaptability and explains how the proposed controller differs fundamentally from conventional MPC and fixed-gain PI/PID strategies^24,25^.

### AMPC tuning procedure

The prediction horizon $\:Np$​, control horizon $\:Nc$, and weighting matrices $\:Q$ and $\:R$ were selected following a structured tuning methodology commonly adopted in AMPC practice^23^. Several candidate combinations were evaluated under step and dynamic load disturbances. The final parameters were selected based on minimizing the ITAE index while ensuring a trade-off between overshoot, settling time, and control effort.

### AMPC integration with LFC

The Adaptive Model Predictive Controller (AMPC) was implemented and integrated into the overall system model using MATLAB/Simulink to ensure real-time regulation and robust tracking performance. The controller operates within a closed-loop configuration, where it continuously receives the measured plant outputs ($\:mo$) and the reference signal (ref) from the system. These signals represent, respectively, the actual plant response and the desired operating point.

An external function block, labeled “Update Plant Model” is responsible for supplying the controller with the time-varying state-space matrices $\:{A}_{K}$,$\:{B}_{k},\:{C}_{k}$,$\:{D}_{k}$ that define the current linearized plant dynamics. This mechanism allows the adaptive MPC to modify its internal prediction model at each control interval according to the instantaneous operating conditions of the plant. The updated model is then used by the controller to compute the optimal manipulated variable ($\:mv$), which drives the system toward the desired reference while minimizing the defined cost function.

The computed control action ($\:mv$) is fed back into the simulated plant subsystem, forming a feedback loop that dynamically compensates for disturbances and parameter variations. The disturbance input and frequency deviation output shown in the Simulink diagram enable continuous monitoring of system performance and facilitate adaptive correction in real time.

This configuration ensures that the Adaptive MPC can maintain system stability and high-quality transient performance even under nonlinear behavior or significant changes in plant characteristics. Compared to a conventional MPC scheme, the adaptive structure provides enhanced robustness and flexibility, as the prediction model is continuously updated to match the true plant dynamics, ensuring accurate state estimation and optimal control performance throughout the simulation.

## Model overview

### Single area power system

**Fig. 1:**
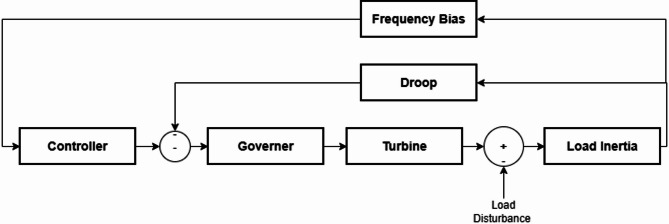
Model of single area system.

The single-area load–frequency control (LFC) loop is shown in Fig. 1. A disturbance in electrical demand (ΔP) enters at the generator–load interface. The measured frequency deviation is processed by a secondary controller whose command is combined with the primary droop contribution before driving the governor. The governor and turbine convert this command into mechanical power, which opposes the disturbance through the generator–load swing dynamics. A frequency-bias term closes the loop to achieve zero steady-state frequency error for step loads while coordinating area control^28^.

In the single-area load–frequency control (LFC) system, the PI controller shapes the closed-loop dynamics and eliminates the steady-state frequency error. The governor converts the control command into a valve position, subject to a first-order lag and practical constraints, while the turbine translates valve motion into mechanical power with an additional lag. A change in electrical load (ΔP) acts as an external disturbance that excites the control loop for disturbance-rejection evaluation. The generator and load dynamics are represented by the swing equation, which couple’s mechanical and electrical power to frequency. The droop characteristic provides a static proportional relationship between frequency and power, enabling power sharing among generating units. Finally, the frequency bias adjusts the measured frequency deviation (Δf) within the Automatic Generation Control (AGC) path to prevent the accumulation of area control errors and ensure stable system operation^4^. The model transfer functions are given in Eqs. (14),(15),(16),(17) & (18) :

14 $$\:\mathrm{G}\mathrm{o}\mathrm{v}\mathrm{e}\mathrm{r}\mathrm{n}\mathrm{o}\mathrm{r}\:\mathrm{m}\mathrm{o}\mathrm{d}\mathrm{e}\mathrm{l}\:=\frac{1}{\left(1\:+\:\mathrm{S}\mathrm{T}\mathrm{g}\right)\:}$$

15 $$\:\mathrm{T}\mathrm{u}\mathrm{r}\mathrm{b}\mathrm{i}\mathrm{n}\mathrm{e}\:\mathrm{m}\mathrm{o}\mathrm{d}\mathrm{e}\mathrm{l}\:=\frac{1}{\left(1\:+\:\mathrm{S}\mathrm{T}\mathrm{t}\right)}\:$$

16 $$\:\mathrm{G}\mathrm{e}\mathrm{n}\mathrm{e}\mathrm{r}\mathrm{a}\mathrm{t}\mathrm{o}\mathrm{r}\:\mathrm{a}\mathrm{n}\mathrm{d}\:\mathrm{l}\mathrm{o}\mathrm{a}\mathrm{d}\:\mathrm{m}\mathrm{o}\mathrm{d}\mathrm{e}\mathrm{l}\:=\frac{1}{\mathrm{M}\mathrm{S}\:+\:\mathrm{D}}$$

17 $$\:\mathrm{D}\mathrm{r}\mathrm{o}\mathrm{o}\mathrm{p}\:=\frac{1}{\mathrm{R}}\:$$

18 $$\:\mathrm{G}\mathrm{o}\mathrm{v}\mathrm{e}\mathrm{r}\mathrm{n}\mathrm{o}\mathrm{r}\:\mathrm{f}\mathrm{r}\mathrm{e}\mathrm{q}\mathrm{u}\mathrm{e}\mathrm{n}\mathrm{c}\mathrm{y}\:\mathrm{b}\mathrm{i}\mathrm{a}\mathrm{s}\:=\:\mathrm{B}.\:\mathrm{D}\:\:$$

Where

R is the speed regulation of the governor.

M is the inertia constant.

B is governor frequency bias.

### Double area power systems

We model two power areas, each with its own controller, governor, turbine, generator-load model and droop/frequency-bias feedback. The areas are linked by a tie-line, so a load step in one area changes the other area’s frequency and the tie-line power.

**Fig. 2:**
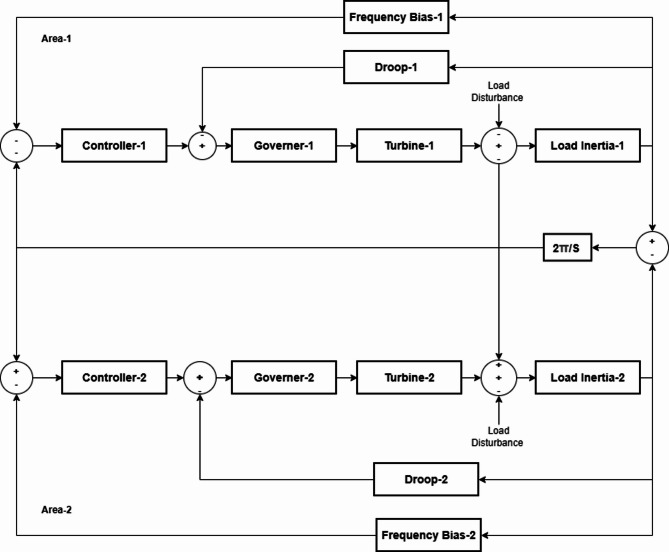
Model of double area system.

 Each controller acts on an Area Control Error (ACE) that blends local frequency deviation with the tie-line error, then drives the governor–turbine to correct power as shown in Fig. 2^4^.

The area errors are shown in Eqs. (19) & (20):

19 $$\:{\mathrm{A}\mathrm{C}\mathrm{E}}_{1}\text{}\approx\:{\mathrm{B}}_{1}\hspace{0.17em}{\Delta\:}{\mathrm{f}}_{1}\hspace{0.25em}\:+\hspace{0.25em}\:{\Delta\:}{\mathrm{P}}_{\mathrm{t}\mathrm{i}\mathrm{e}}\:$$

20 $$\:{\mathrm{A}\mathrm{C}\mathrm{E}}_{2}\text{}\:\approx\:\:{\mathrm{B}}_{2}\hspace{0.17em}{\Delta\:}{\mathrm{f}}_{2}\hspace{0.25em}\:-\hspace{0.25em}\:{\Delta\:}{\mathrm{P}}_{\mathrm{t}\mathrm{i}\mathrm{e}}$$

## Simulation and results

In this paper, the proposed Adaptive model predictive controller (AMPC) is subject to test its effectiveness in a single area power system and double area power system against different controllers. As, there are three case studies related to single area power system as following, the first one is A step-load disturbance of 0.1 p.u, the second is a dynamic load disturbance and the third is wind generation as a disturbance, and four case studies for the double area power system as following, the first one is 1% load change to Area 1, the second is 1% load change to Area 2, the third is a dynamic load disturbance to Area 1 and the final one is a dynamic load disturbance to Area 2.

### Single area power system

This section discusses in detail the single area power system parameters and the three different case studies, two case studies without wind disturbance and one case study with wind disturbance. Tables 1 and 2 illustrate the single area system parameters and Best-Fit Controller Gains.

**Table 1:** Single-area system Parameters.

Parameter/Gains	Single Area Parameters
Tg	0.3s
Tt	0.1s
R	0.05
B	21
D	1
M	10s

**Table 2:** Single-area system Best-Fit controller Gains.

Parameter/Gains	Case1 Gains	Case2 Gains	Case3 Gains
Kp(PI)	0.45	0.41	0.41
Ki(PI)	0.32	0.28	0.28

#### Results of case study#1

**Fig. 3:**
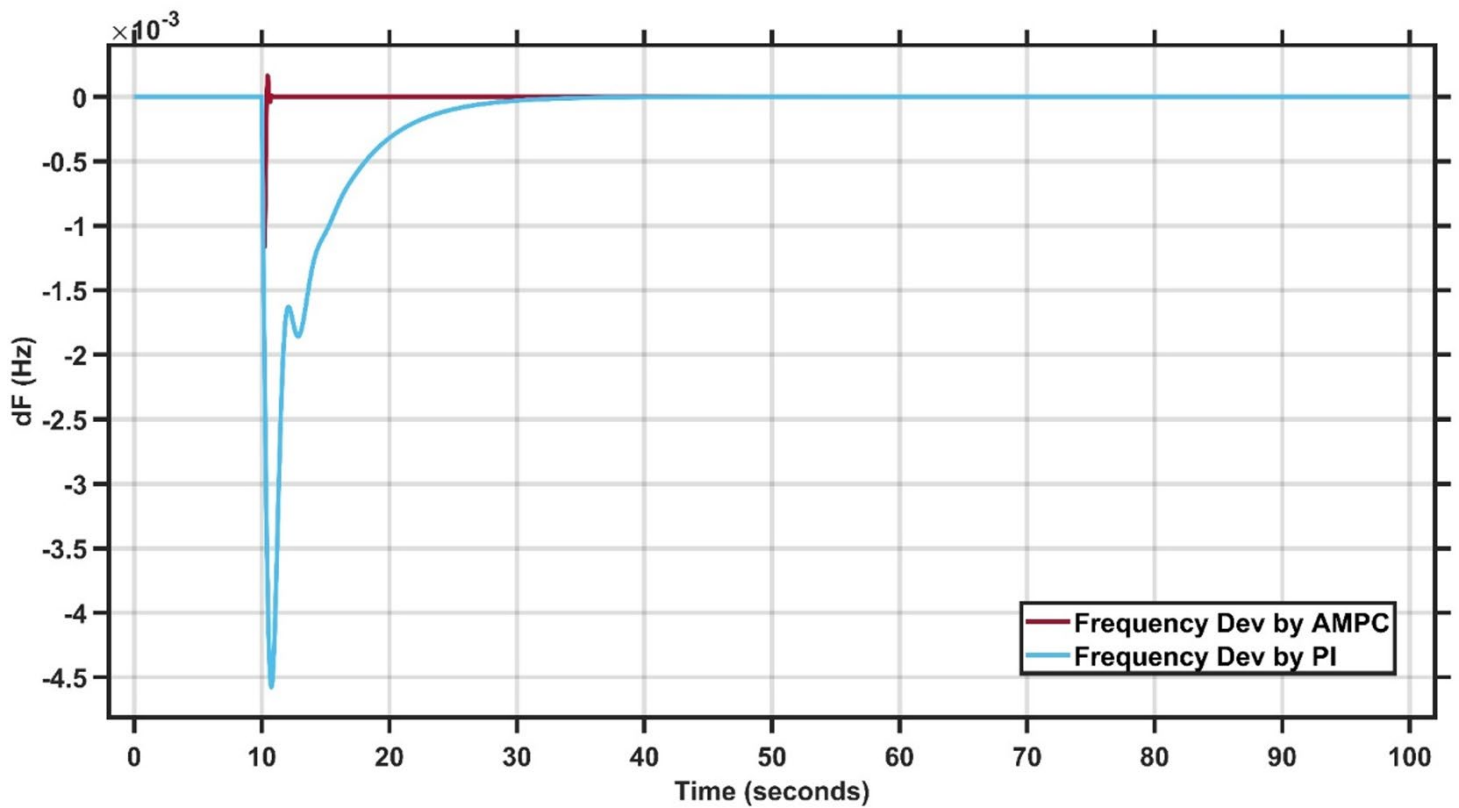
Change in frequency between AMPC and PI controller for case study 1.

The system is tested using two controllers: the conventional PI controller and the proposed Adaptive Model Predictive Controller (AMPC). A step-load disturbance of 0.1 p.u. is applied to evaluate their performance. The PI controller gains, compared with those reported in^27^, show a high degree of consistency, confirming the robustness and accuracy of the simulation, as summarized in Tables 1 and 2, which presents the best-fit gains for Case Study 1. As illustrated in Fig. 3, the system initially operates at steady state, after which the load change occurs. The AMPC demonstrates superior dynamic performance compared to the PI controller, achieving faster recovery and smoother response. Notably, the AMPC effectively eliminates oscillations and significantly reduces the settling time, allowing the system to reach steady state more efficiently. These results confirm the superior effectiveness of the proposed AMPC controller for this case study. The transient response specifications of the AMPC and PI controllers for case study #1 are summarized in Table 3.

**Table 3:** Transient response specifications of case study #1.

Variables	AMPC	PI Controller
Overshoot	≈ 0	0
Undershoot	−1 × 10⁻³	–4.5 × 10⁻³
Settling Time	≈ 0.5–1 s	30 s

#### Results of case study#2

**Fig. 4:**
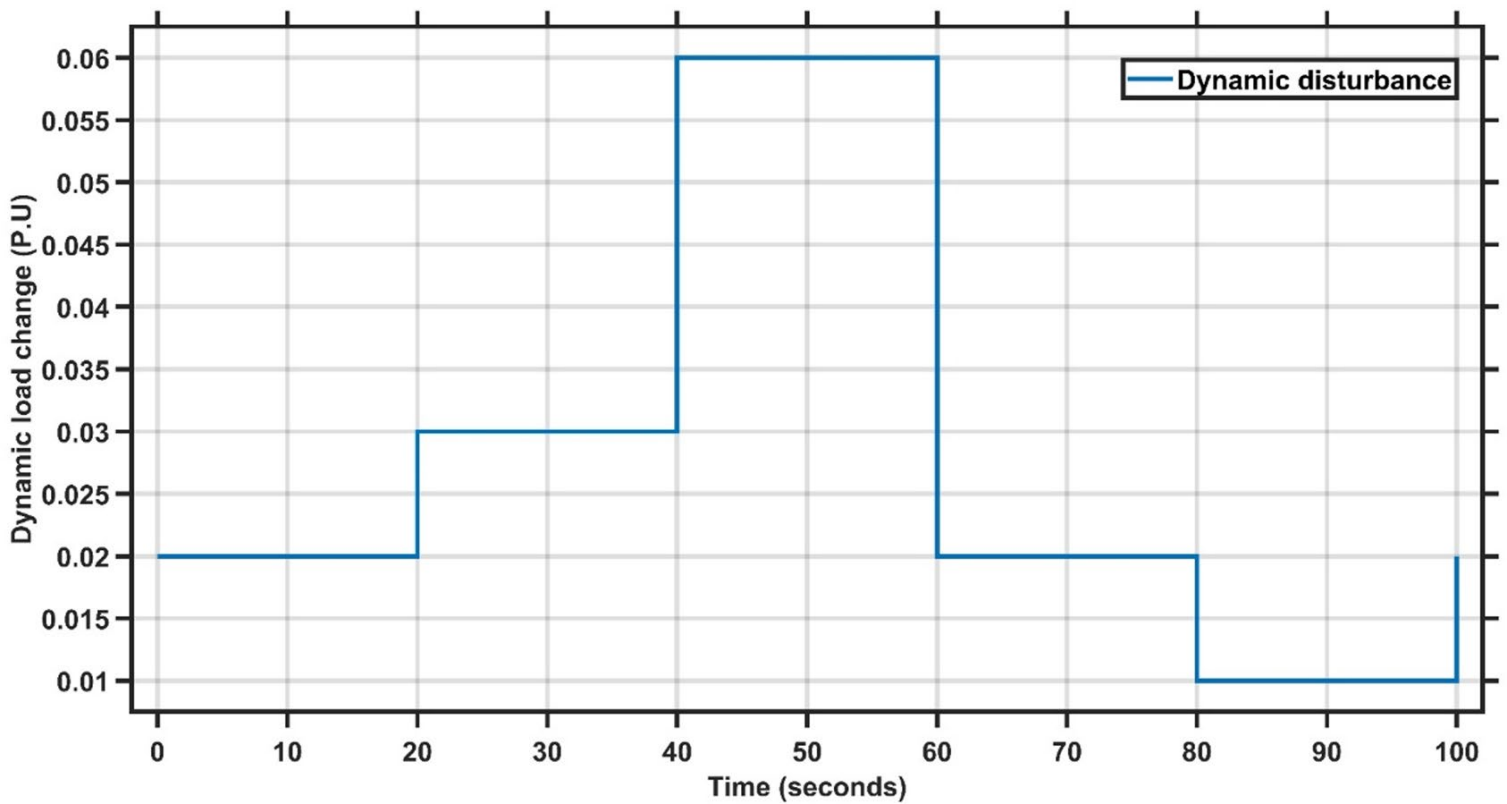
Dynamic disturbance for case study 2.

**Fig. 5:**
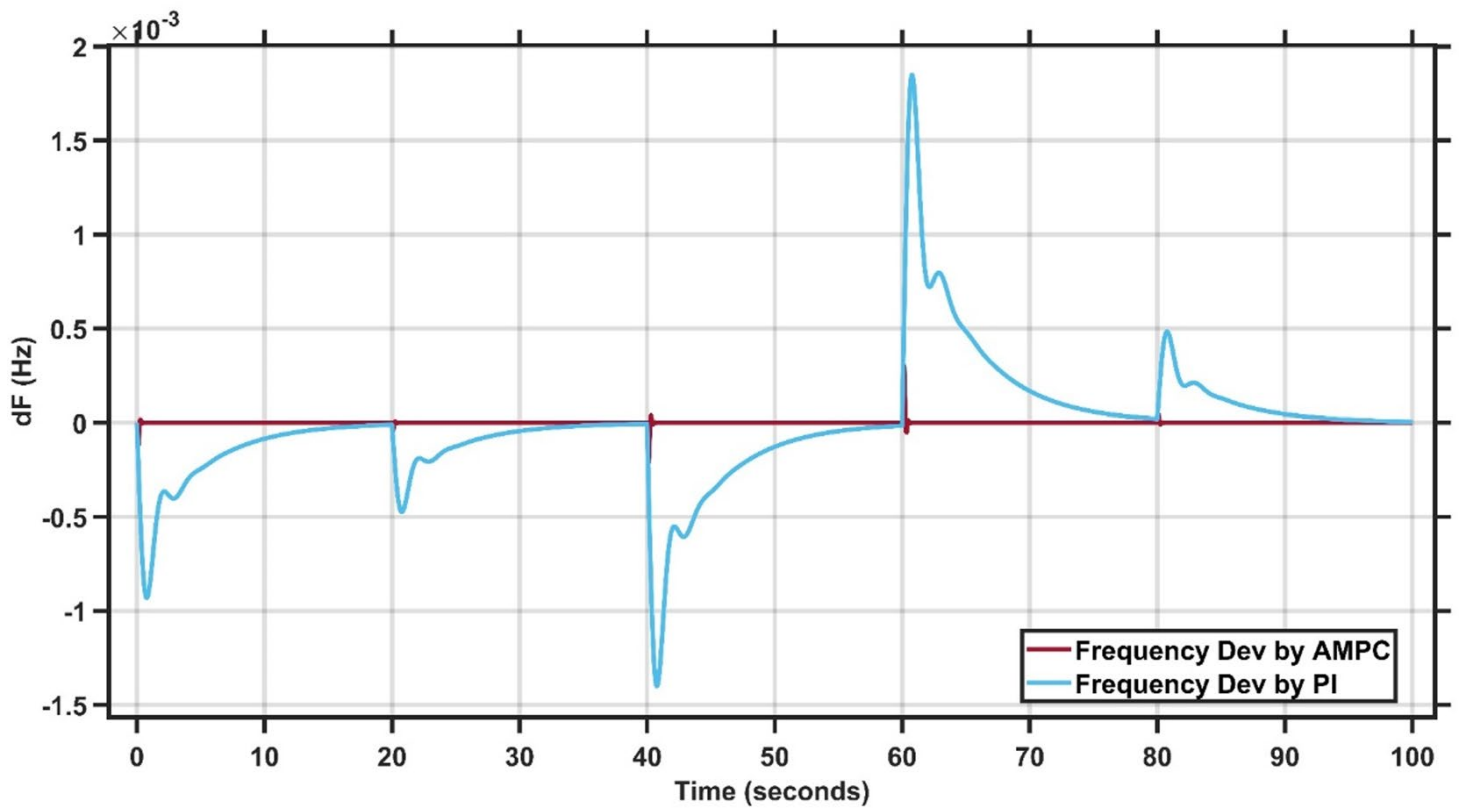
Change in frequency between AMPC and PI controller for case study 2.

Case Study 2 is similar to Case Study 1, using the same system parameters but subjected to a dynamic load disturbance, as illustrated in Fig. 4. Table 1 presents the best-fit gains for the PI controller, while Fig. 5 clearly demonstrates that the proposed AMPC controller significantly outperforms the conventional PI controller. The overshoot is almost completely eliminated when using the AMPC, and the system reaches steady state much faster. These findings confirm that the AMPC controller delivers superior dynamic performance and enhanced frequency stability compared to the PI controller. The transient response specifications of the AMPC and PI controllers for case study #2 are summarized in Table 4.

**Table 4:** Transient response specifications of case study #2.

Variables	AMPC	PI Controller
Maximum Peak to Peak shoot	≈ 0	3.1 × 10⁻³
Settling Time	≈ 0 s	20 s

#### Results of case study#3

**Fig. 6:**
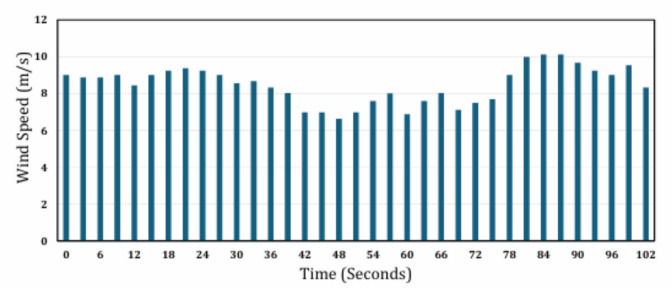
Variation of wind speed with time.

Case Study 3 uses the same single-area power system and parameters as in the previous cases, with the addition of wind generation as a disturbance. The wind generator has a swept area of 5538.96 m² and a power coefficient $\:{C}_{p}=0.5$. The wind power generated is calculated using the following Eq. (21):

21 $$\:P=\frac{1}{2}\rho\:{C}_{p}A{V}^{3}$$

where $\:\rho\:=1.225{\hspace{0.17em}}{\mathrm{kg/m}}^{3}$ is the air density, $\:{C}_{p}$ is the power coefficient, $\:A$is the swept area (m²), and $\:V$ is the wind speed (m/s). The variation of wind speed over time is illustrated in Fig. 6, as presented in^28^.

**Fig. 7:**
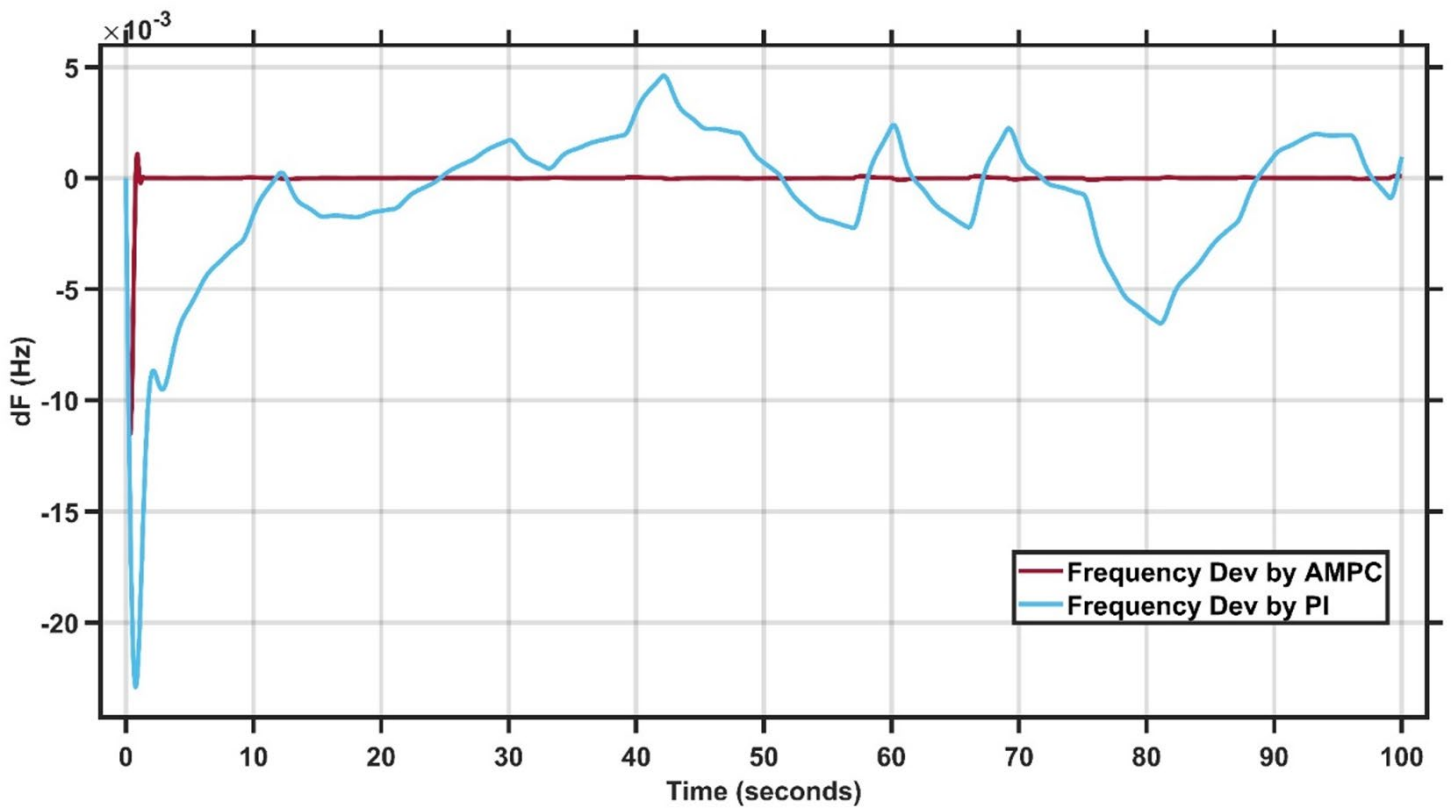
Change in frequency between AMPC and PI controller for case study 3.

Table 1 presents the best-fit gains for the three controllers, while Fig. 7 illustrates that the proposed AMPC controller demonstrates superior performance and robustness compared to the conventional PI controller under wind disturbance conditions. The AMPC achieves a significantly smaller undershoot, reduced oscillations with lower peak amplitudes, and a much faster settling time. These results clearly confirm the enhanced capability of the AMPC controller in effectively managing renewable energy–induced disturbances and maintaining system stability. The transient response specifications of the AMPC and PI controllers for case study #3 are summarized in Table 4.

**Table 4:** Transient response specifications of case study #3.

Variables	AMPC	PI Controller
Maximum Peak to Peak shoot	≈ 0	26.5 × 10⁻³
Steady state error	≈ 0 s	1 × 10⁻³

### Double area power system

This section analyzes the double-area power system, which consists of two single-area systems interconnected through tie-lines, as illustrated in Fig. 2. Each area operates with its own set of parameters and Best-Fit Controller Gains, as listed in Tables 5 and 6. The proposed AMPC controller is evaluated and compared with the conventional PID controller^28^ under identical load-change conditions. The simulation duration is set to 60 s to assess the system’s steady-state performance before, during, and after the load disturbance, enabling a comprehensive comparison of the dynamic and steady-state responses of both controllers.

**Table 5:** Double-area system Parameters.

Parameter/Gain	System Parameters	
	Area1	Area2
Tt(s)	0.5	0.6
Tg (s)	0.2	0.3
R	0.05	0.0625
B	20.6	16.9
D	0.6	0.90
M(s)	10	8

**Table 6:** Double-area system Best-Fit controller Gains.

Parameter/Gain	1% Load Change at Area1		1% Load Change at Area2	
	Area1	Area2	Area1	Area2
Kp	0.35	0.28	0.61	0.47
Ki	0.22	0.7	0.3	0.38
Kd	0.44	0.48	0.28	0.12

#### Case study 1–1% load change at area 1

**Fig. 8:**
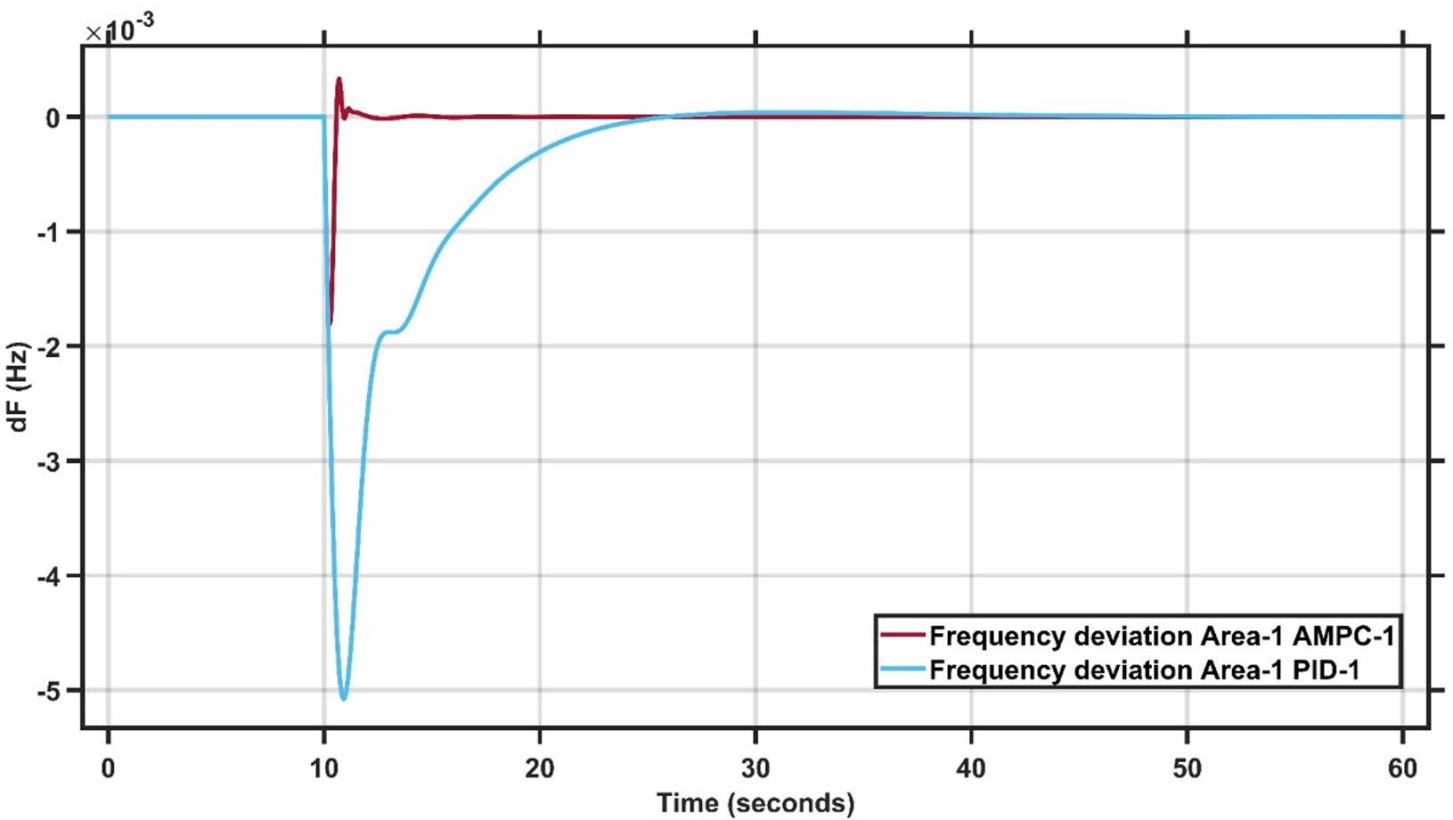
Frequency output of area 1 at 1% load change in area 1.

**Fig. 9:**
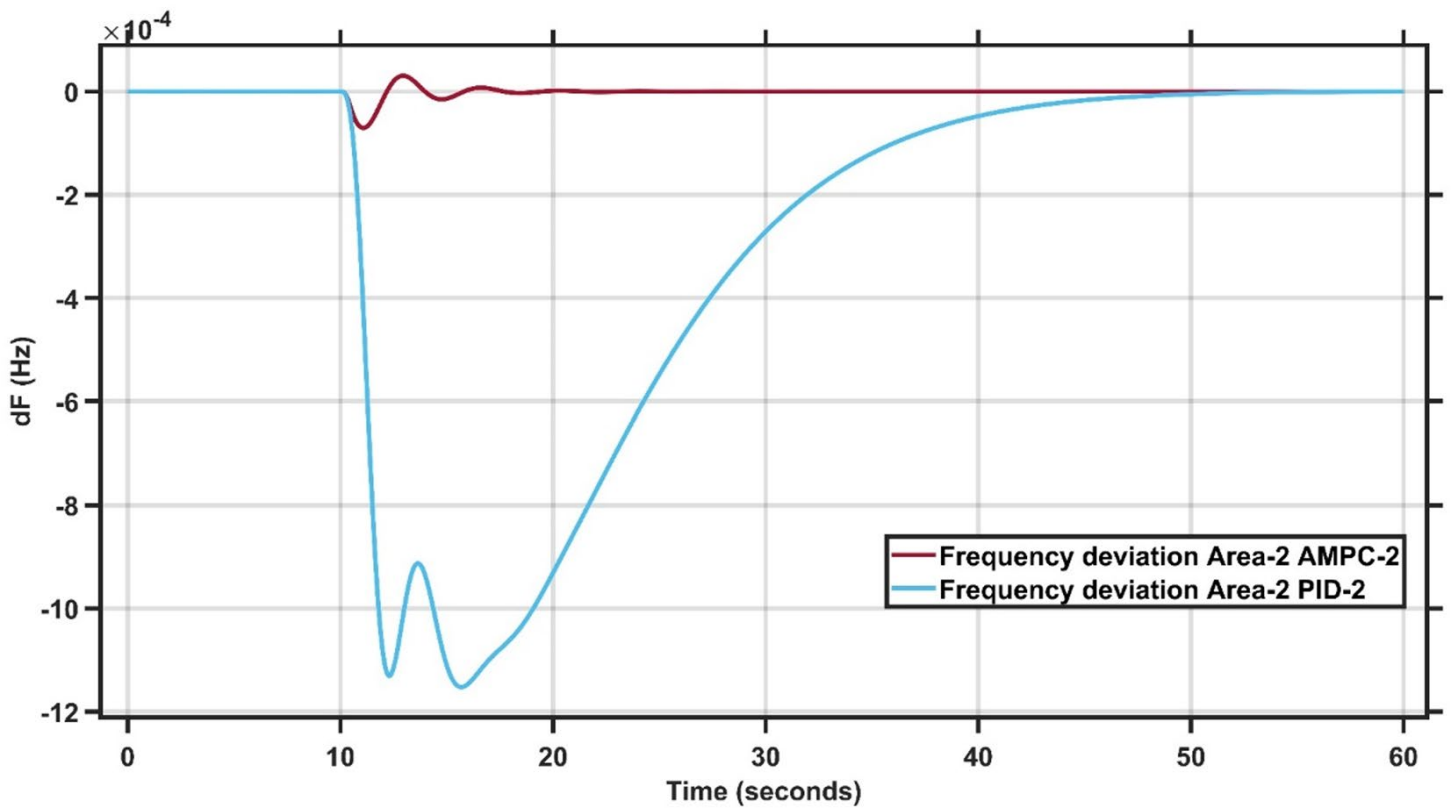
Frequency output of area 2 at 1% load change in area 1.

Case Study 1 of the double-area power system applies a 1% load change to Area 1, while Area 2 remains unaffected. The results for this scenario are summarized in Table 2. Figures 8 and 9 present the frequency responses of the proposed AMPC controller compared with the conventional PID controller for Areas 1 and 2, respectively. The PID controller gains were obtained through a trial-and-error tuning process. As shown in Fig. 8, the system operates at steady state before the disturbance, and following the load change, the AMPC controller exhibits superior dynamic performance compared to the PID controller. Specifically, the AMPC achieves a smaller undershoot and a faster settling time. Figure 9 further confirms that the frequency response in Area 2 also improves when the AMPC controller is employed, demonstrating its effectiveness in enhancing inter-area stability. The transient response specifications of the AMPC and PID controllers for case study #1 are summarized in Table 7.

**Table 7:** Transient response specifications of case study #1.

Variables	Area-1		Area-2	
	AMPC	PID	AMPC	PID
Overshoot	0.4 × 10⁻³	0	0.8 × 10⁻⁴	0
Undershoot	−0.3 × 10⁻³	−4.8 × 10⁻³	−1.2 × 10⁻⁴	−11.2 × 10⁻⁴
Settling Time	≈ 1 s	≈ 23 s	≈ 1 s	≈ 38 s

#### Case study 2 − 1% load change at area 2

**Fig. 10:**
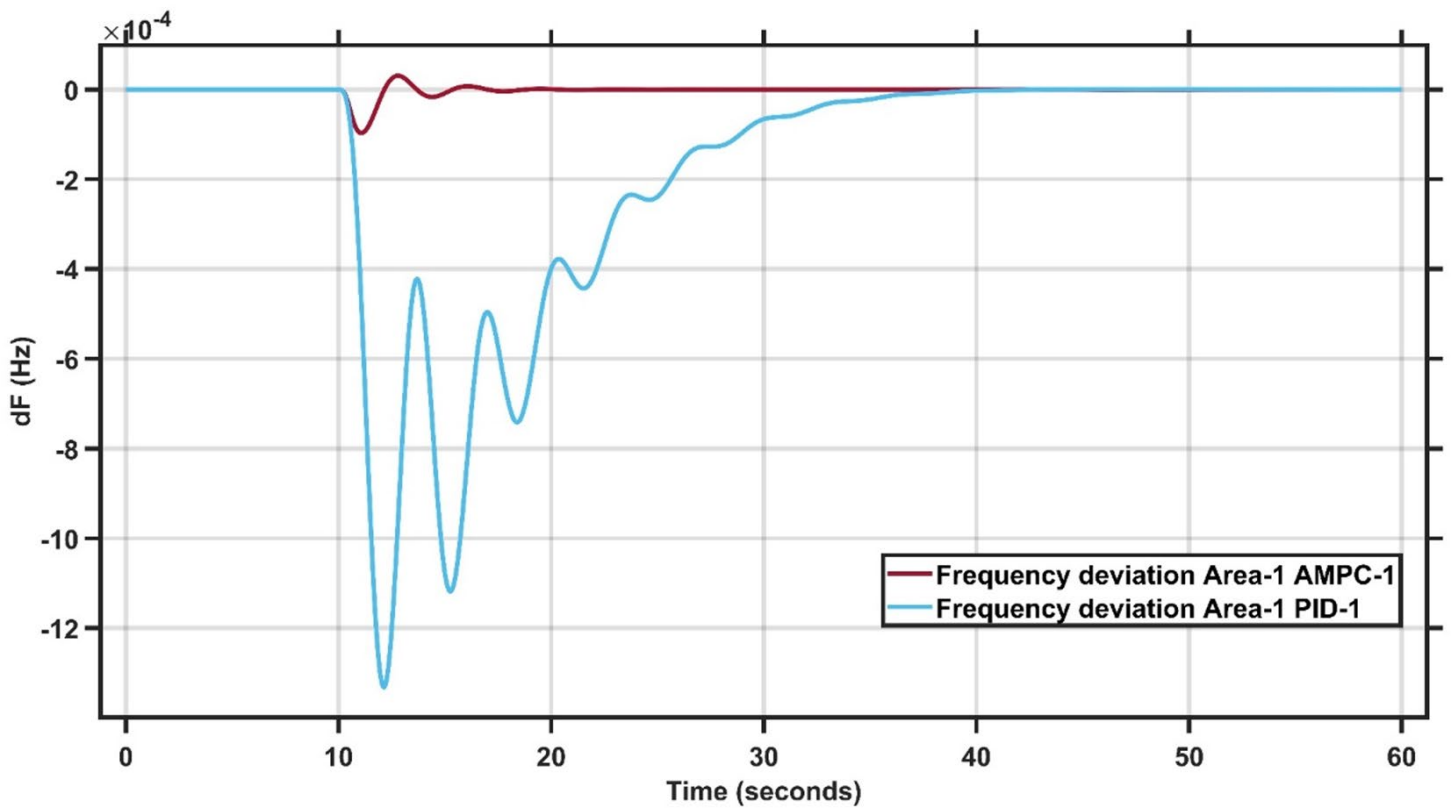
Frequency output of area 1 at 1% load change in area 2.

**Fig. 11:**
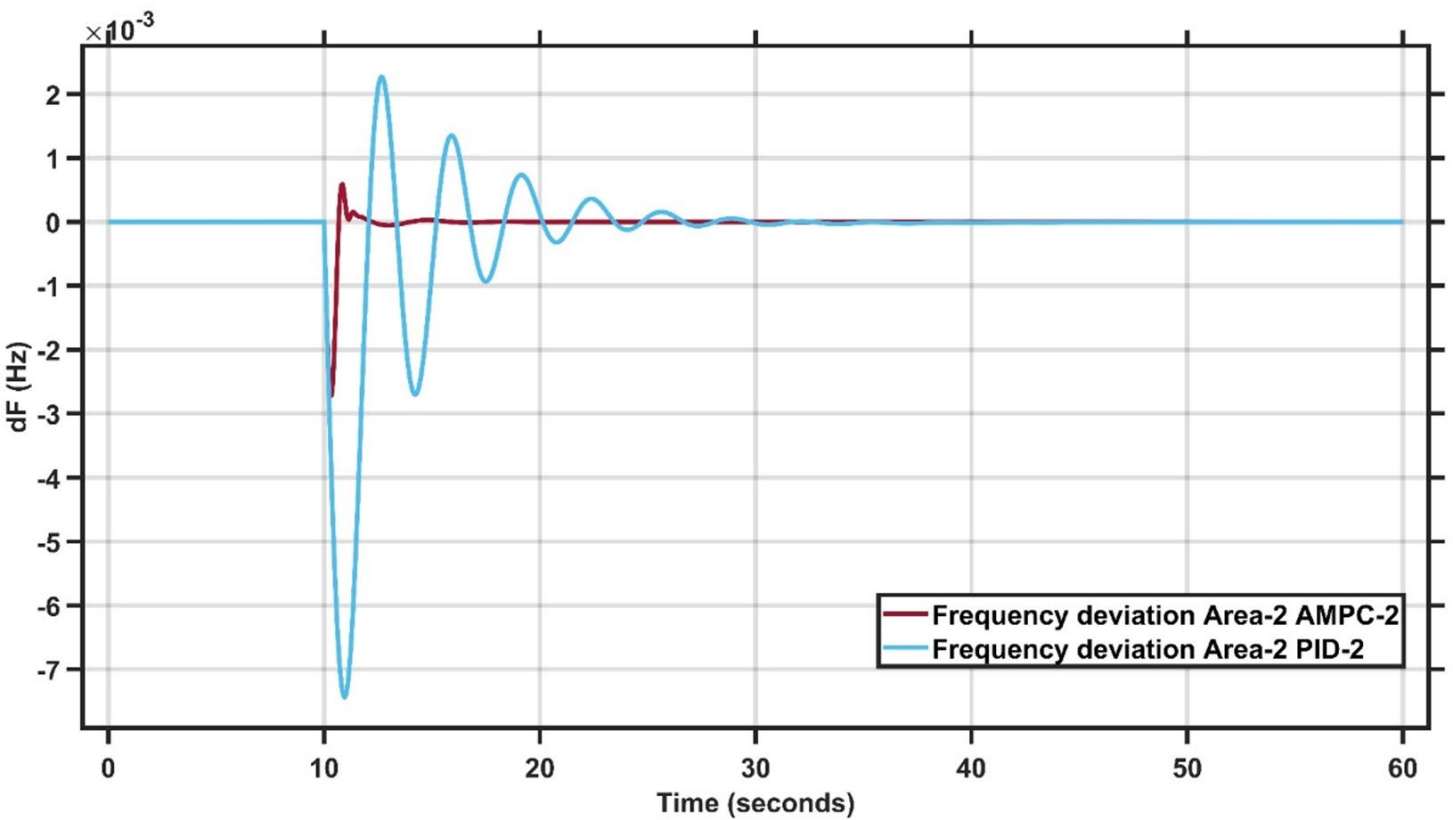
Frequency output of area 2 at 1% load change in area 2.

In Case Study 2, the load change is applied to Area 2 instead of Area 1. The optimal gains and initial parameter values for the controllers are listed in Table 2. Figures 10 and 11 compare the frequency responses of the proposed AMPC controller with those of the conventional PID controller. As shown in Fig. 10, the frequency response in Area 1 indicates that the AMPC controller achieves a significantly faster settling time, with minimal oscillations and a smaller undershoot than the PID controller. Similarly, Fig. 11 shows the frequency response in Area 2, where the AMPC controller attains a rapid and smooth recovery with almost no oscillations from the onset of the load disturbance. The frequency undershoot is consistently lower than that of the PID controller, and steady-state conditions are reached more quickly, confirming the superior performance of the AMPC approach. The transient response specifications of the AMPC and PID controllers for case study #2 are summarized in Table 8.

**Table 8:** Transient response specifications of case study #2.

Variables	Area-1		Area-2	
	AMPC	PID	AMPC	PID
Overshoot	0.6 × 10⁻⁴	0	0.5 × 10⁻³	2.0 × 10⁻³
Undershoot	−1.0 × 10⁻⁴	−12 × 10⁻⁴	−0.8 × 10⁻³	−7.0 × 10⁻³
Settling Time	≈ 1–2 s	≈ 35–40 s	≈ 1–2 s	≈ 20–22 s

#### Case study 3 - dynamic load change at area 1

**Fig. 12:**
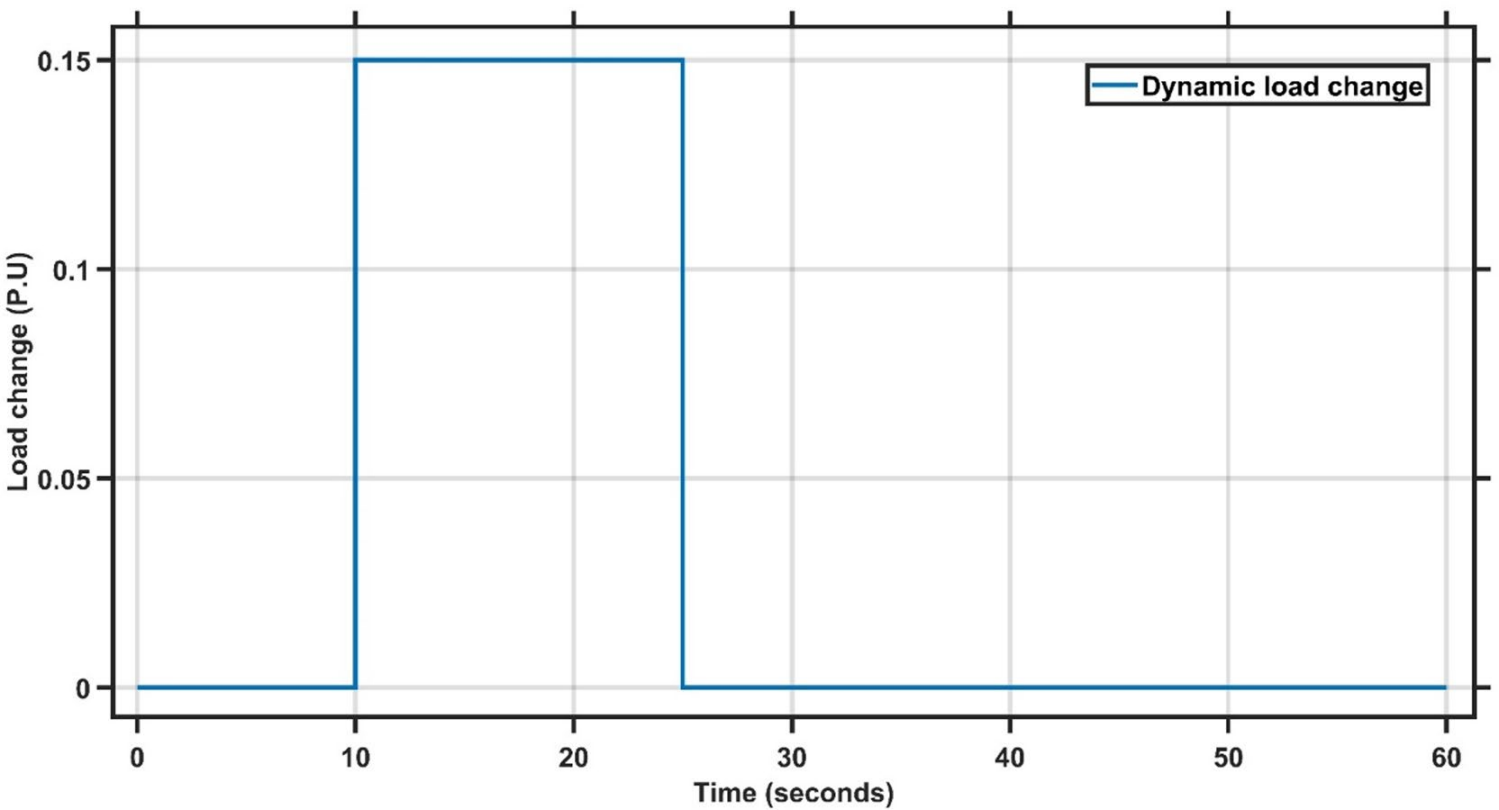
Dynamic load change.

**Fig. 13:**
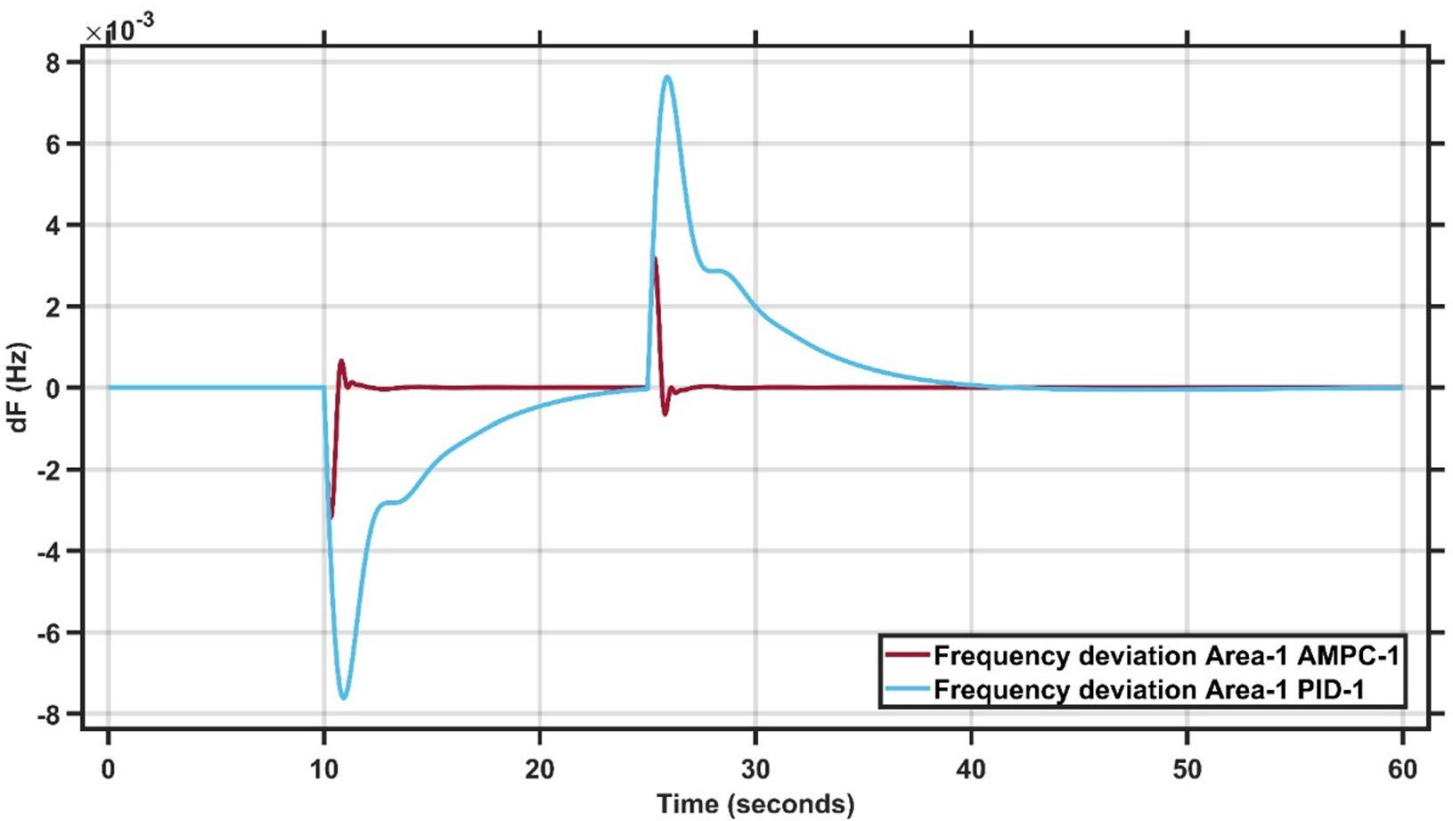
Frequency output of area 1 at dynamic load change in area 1.

**Fig. 14:**
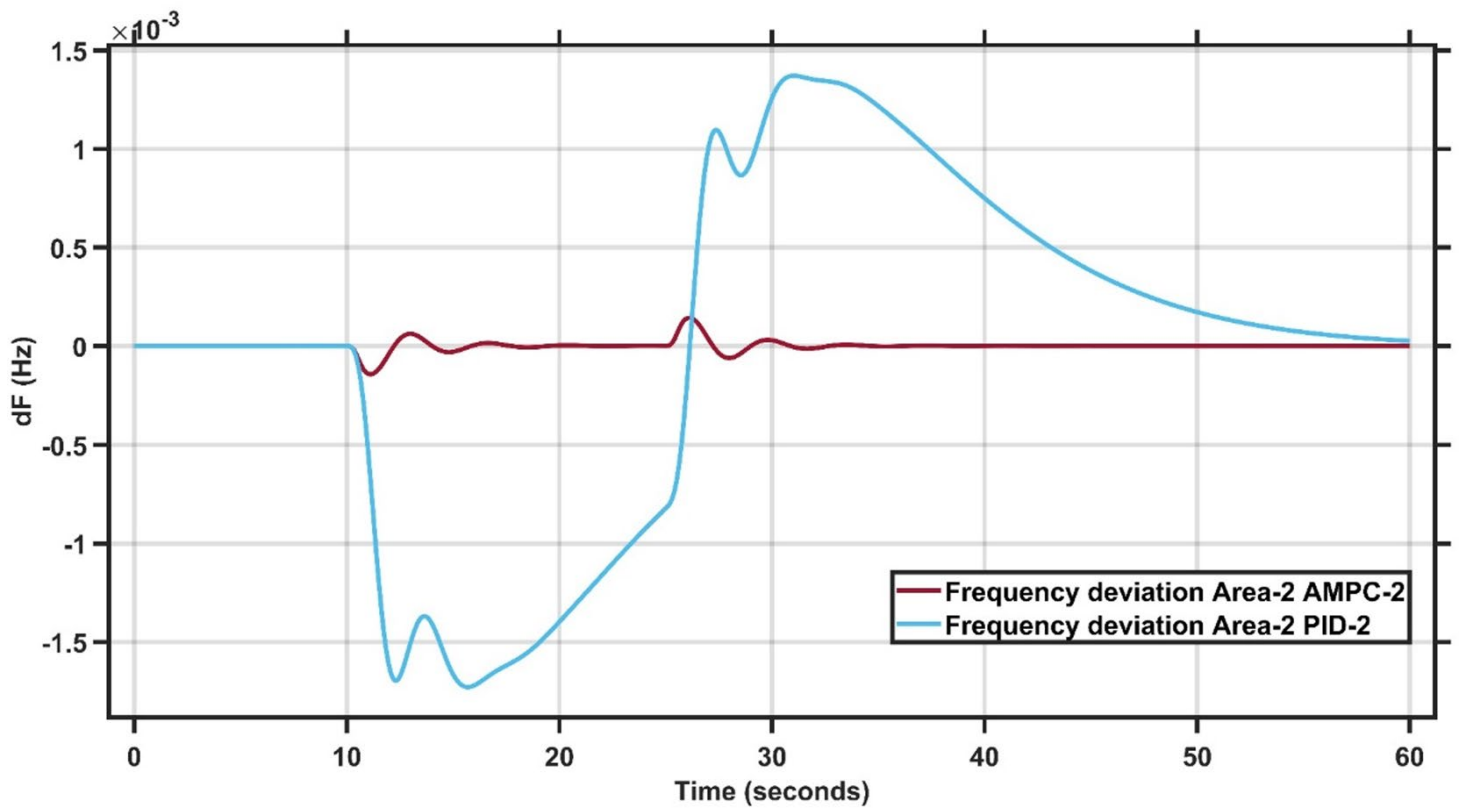
Frequency output of area 2 at dynamic load change in area 1.

Case Study 3 follows the same conditions as Case Study 1 but replaces the step load change of 0.1 p.u. with a dynamic load disturbance. As illustrated in Fig. 12, the load remains zero from $\:t=0\:$to $\:t=10$seconds, increases by 15% between $\:t=10\:$and $\:t=25\:$seconds, and then returns to zero from $\:t=25\:$to $\:t=60\:$seconds. The double-area power system is tested using both the AMPC and PID controllers. As shown in Fig. 13, the AMPC controller outperforms the PID controller by achieving a faster settling time, lower overshoot and undershoot, and a quicker return to steady state. Figure 14 further emphasizes this improvement, revealing that the AMPC controller maintains the frequency deviation close to zero with minimal oscillations. Overall, the results confirm the superior dynamic performance and stability of the AMPC controller compared to the conventional PID controller. The transient response specifications of the AMPC and PID controllers for case study #3 are summarized in Table 9.

**Table 9:** Transient response specifications of case study #3.

Variables	Area1		Area2	
	AMPC	PID	AMPC	PID
Maximum Peak to Peak shoot	5 × 10⁻³	1.5 × 10⁻²	0.8 × 10⁻³	2.9 × 10⁻³
Settling Time	≈ 1–2 s	≈ 15–20 s	≈ 1–5 s	≈ 40 s

#### Case study 4 - dynamic load change at area 2

**Fig. 15:**
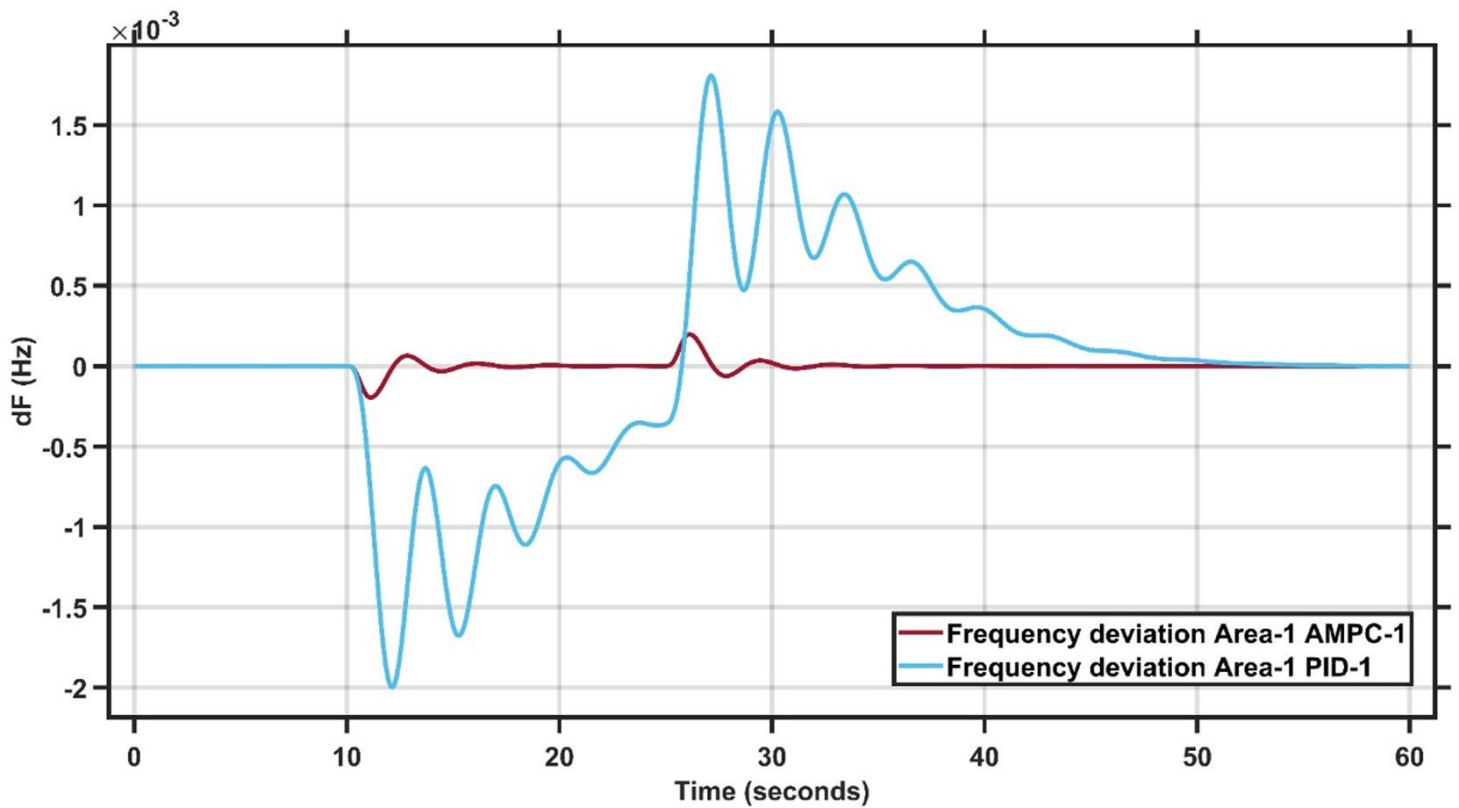
Frequency output of area 1 at dynamic load change in area 2.

**Fig. 16:**
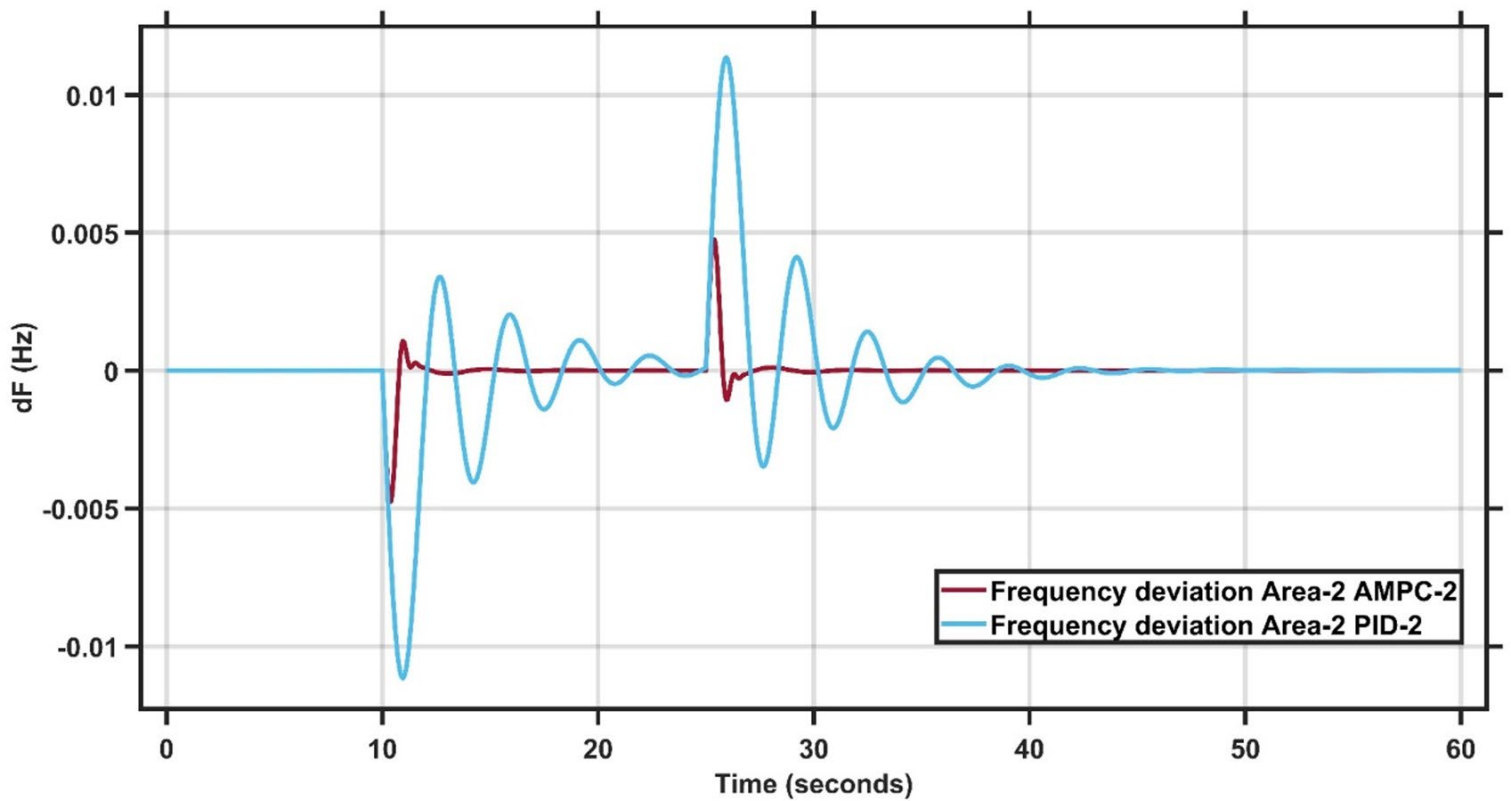
Frequency output of area 2 at dynamic load change in area 2.

In Case Study 4, the dynamic load change is applied to Area 2 instead of Area 1. Figure 15 clearly demonstrates the superior performance of the AMPC controller, as the frequency output remains nearly steady throughout the simulation, whereas the PID controller exhibits noticeable oscillations and struggles to reach steady state. Similarly, Fig. 16 reinforces this finding, showing that the AMPC controller provides a more stable and responsive frequency behavior compared to the PID controller. Overall, Figs. 15 and 16 confirm the effectiveness of the proposed AMPC approach, which achieves steady state significantly faster and with minimal oscillations, ensuring a more stable and reliable frequency response than the conventional PID controller. The transient response specifications of the AMPC and PID controllers for case study #4 are summarized in Table 10.

**Table 10:** Transient response specifications of case study #4.

Variables	Area1		Area2	
	AMPC	PID	AMPC	PID
Maximum Peak to Peak shoot	0.35 × 10⁻³	3.6 × 10⁻³	1 × 10⁻²	2.2 × 10⁻²
Settling Time	≈ 1–5 s	≈ 30 s	≈ 1–5 s	≈ 25–30 s

### Robustness of AMPC

#### Robustness of AMPC against PID controller with HS, SCA & TLBO optimizations

**Fig. 17:**
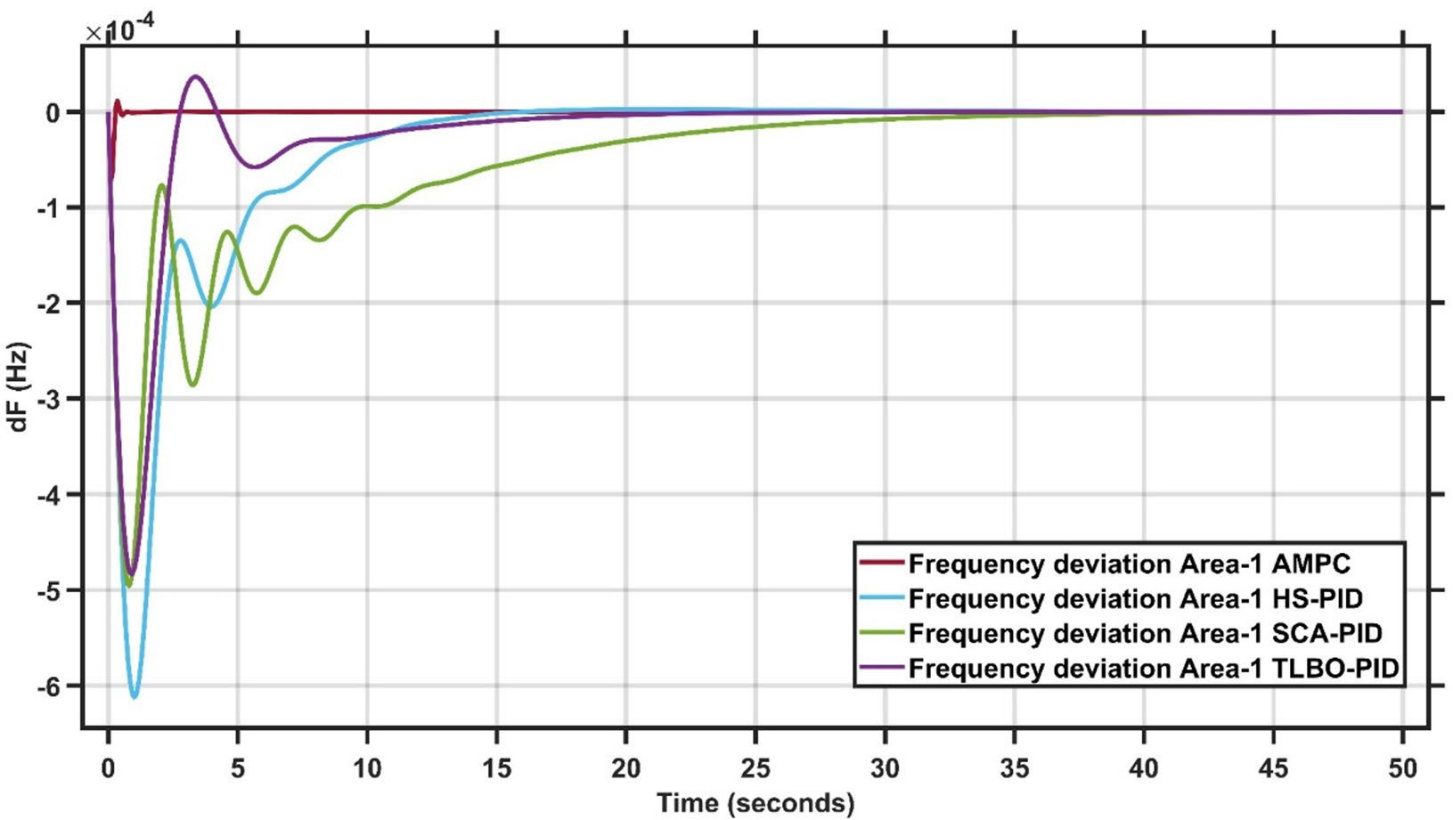
Frequency output of area 1 at 1% load disturbance in area 1&2.

**Fig. 18:**
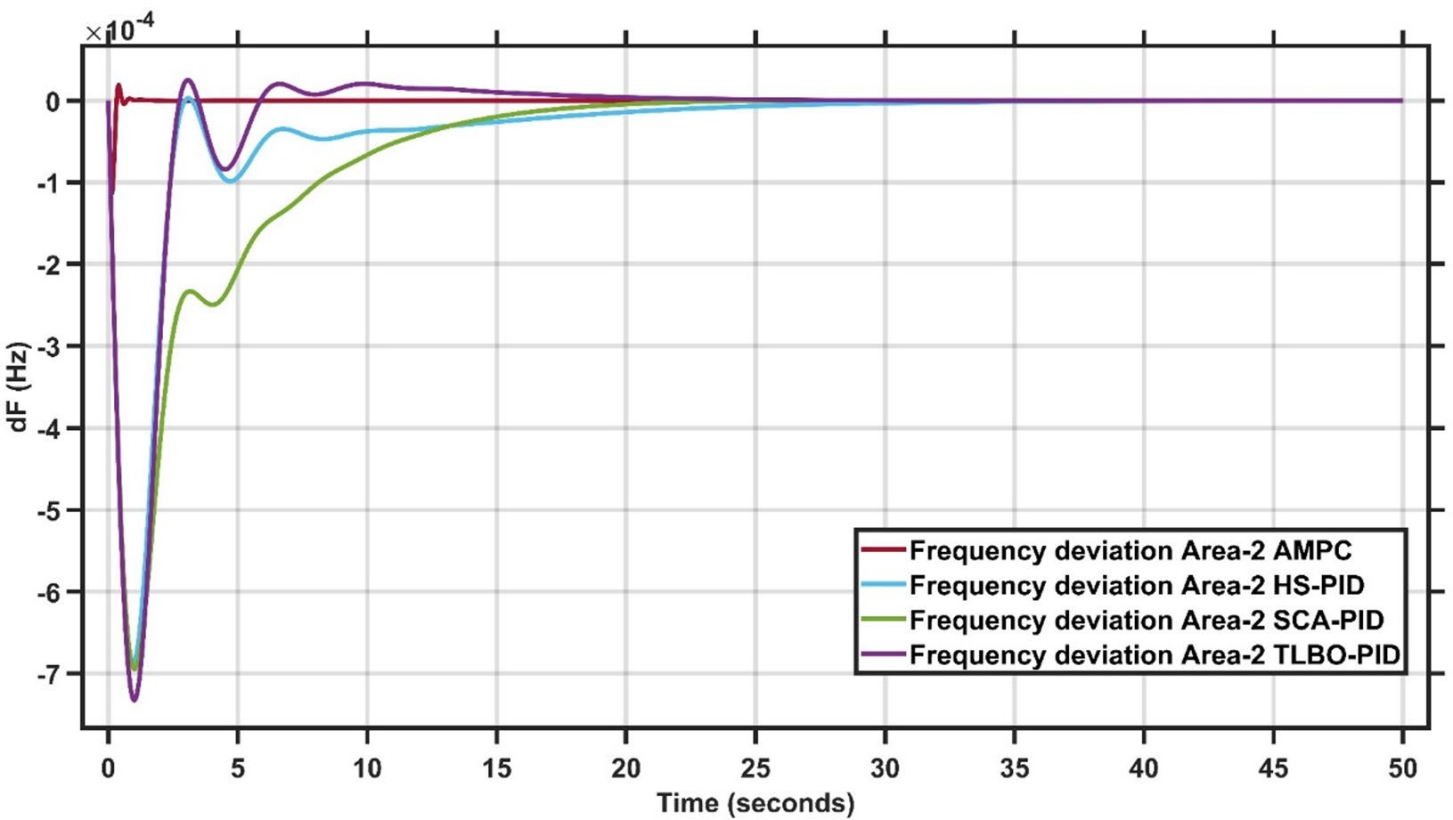
Frequency output of area 2 at 1% load disturbance in area 1&2.

In this section, 1% load disturbance is applied to Area 1 & Area 2. Figures 17 and 18 clearly illustrates the proposed AMPC controller consistently outperforms all optimized PID-based controllers by providing lower peak deviations, faster oscillation damping, and shorter settling times in both areas. Unlike HS-PID, SCA-PID, and TLBO-PID which exhibit larger transient excursions and slower recovery. The AMPC maintains a smoother, more stable response with minimal overshoot and undershoot. Its superior disturbance-rejection capability and robustness across the two areas highlight its effectiveness in achieving coordinated, high-precision frequency regulation^29^.

It is clear that the proposed AMPC controller has the faster settling time around 2 s compared to PID HS, SCA and TLBO based which has around 15 s, 20 s and 35 s respectively in Area (1) And also, AMPC controller has the faster settling time around 2 s compared to PID HS, SCA and TLBO based which has around 30 s, 20 s and 25 s respectively in Area (2) Regarding overshoot AMPC controller and PID HS, SCA and TLBO based all of them nearly don’t have overshoot in Area (1) And also, AMPC controller and PID HS, SCA and TLBO based all of them nearly don’t have overshoot in Area (2) regarding undershoot AMPC controller has the lowest undershoot around 0.7 × 10^− 4^ Hz compared to PID HS, SCA and TLBO based which has around 6.2 × 10^− 4^ Hz, 5 × 10^− 4^ Hz and 4.8 × 10^− 4^ Hz respectively in Area (1) And also AMPC controller has the lowest undershoot around 1.2 × 10^− 4^ Hz compared to PID HS, SCA and TLBO based which has around 6.2 × 10^− 4^ Hz, 6.9 × 10^− 4^ Hz and 7.4 × 10^− 4^ Hz respectively in Area (2) And also AMPC controller has the lowest oscillations compared to PID HS, SCA and TLBO based in Area 1&2.

#### Robustness of AMPC against PIDA controller with HS, SCA & TLBO optimizations

**Fig. 19:**
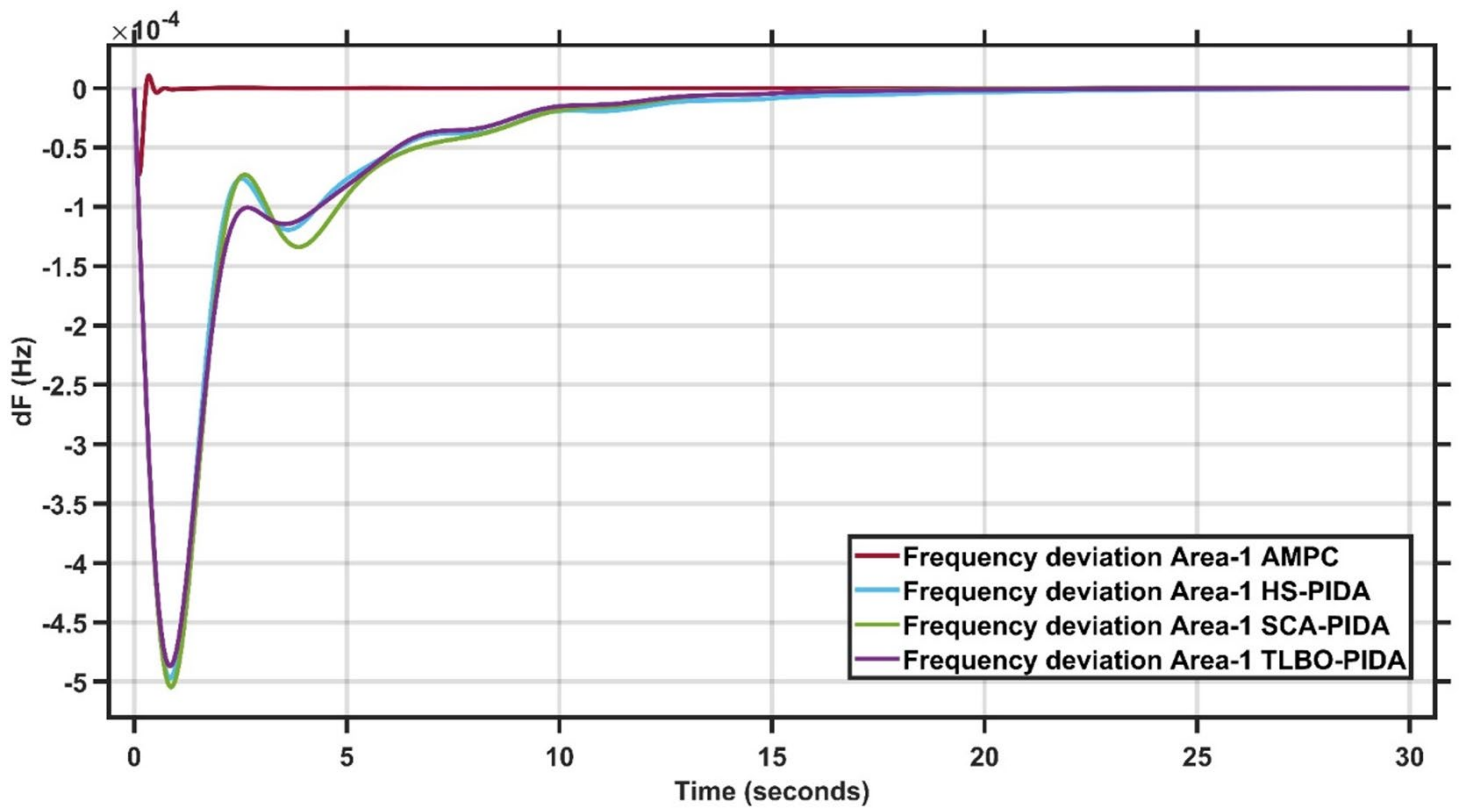
Frequency output of area 1 at 1% load disturbance in area 1&2.

**Fig. 20:**
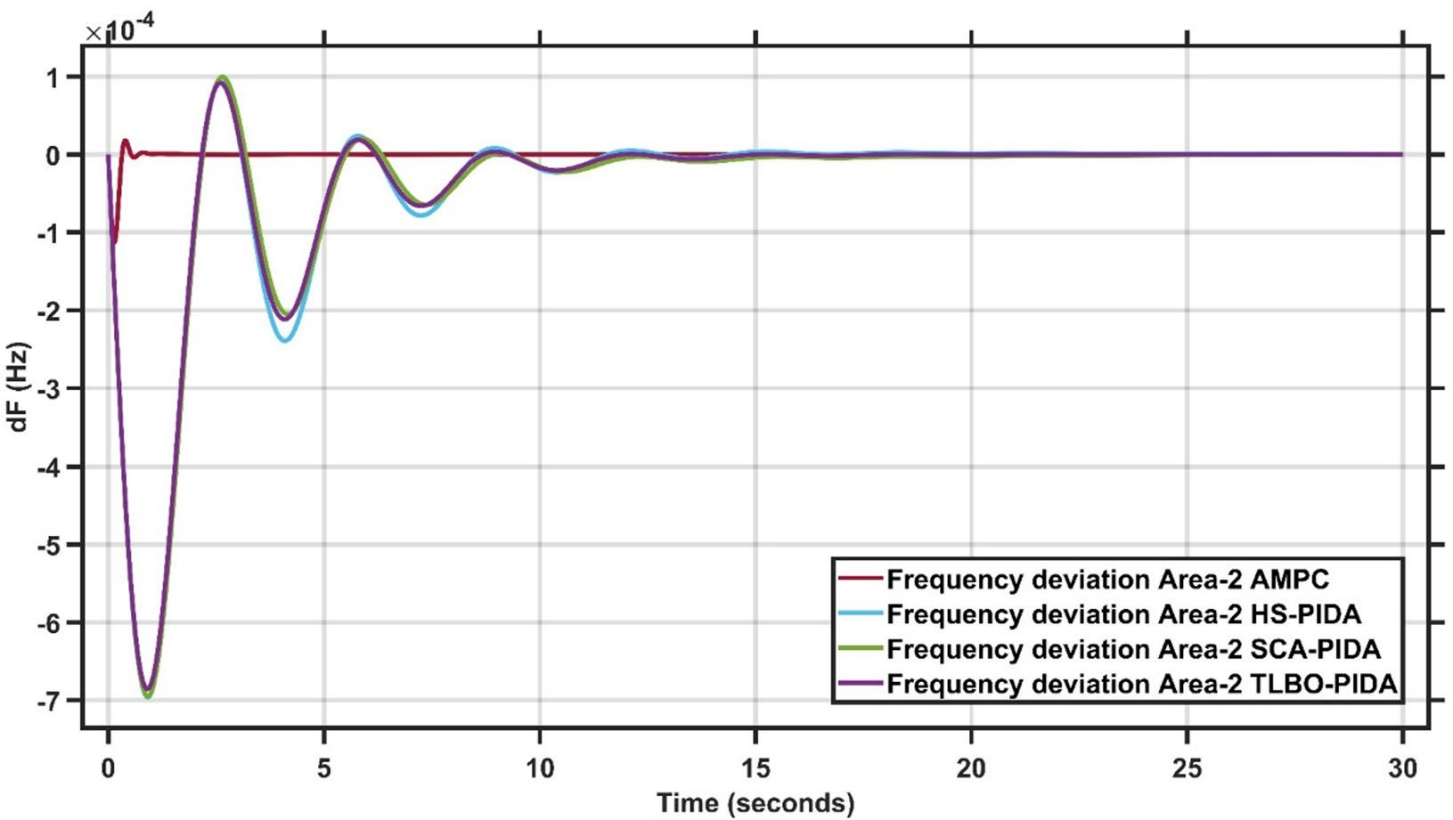
Frequency output of area 2 at 1% load disturbance in area 1&2.

In this section, 1% load disturbance is applied to Area 1 & Area 2. Figures 19 and 20 clearly illustrates across both areas, the proposed AMPC controller demonstrates clear superiority over the optimized PIDA-based controllers. AMPC achieves markedly smaller peak deviations, faster damping of oscillations, and a significantly shorter settling time. While HS-PIDA, SCA-PIDA, and TLBO-PIDA exhibit noticeable oscillatory behavior and slower recovery, the AMPC response remains smooth, stable, and non-oscillatory. These consistent improvements highlight the enhanced robustness and disturbance-rejection capability of the AMPC strategy in multi-area frequency regulation^30^.

It is clear that the proposed AMPC controller has a faster settling time around 1 s compared to PIDA HS, SCA and TLBO based which has around 20 s, 20 s and 20 s respectively in Area (1) And also, AMPC controller has the faster settling time around 2 s compared to PIDA HS, SCA and TLBO based which has around 15 s, 15 s and 15 s respectively in Area (2) Regarding overshoot AMPC controller and PIDA HS, SCA and TLBO based all of them nearly don’t have overshoot in Area (1) And also AMPC controller has the lowest overshoot around 0.2 × 10^− 4^ Hz compared to PIDA HS, SCA and TLBO based which has around 1 × 10^− 4^ Hz, 1 × 10^− 4^ Hz and 1 × 10^− 4^ Hz respectively in Area (2) regarding undershoot AMPC controller has the lowest undershoot around 0.7 × 10^− 4^ Hz compared to PIDA HS, SCA and TLBO based which has around 5 × 10^− 4^ Hz, 5 × 10^− 4^ Hz and 4.9 × 10^− 4^ Hz respectively in Area (1) And also AMPC controller has the lowest undershoot around 1 × 10^− 4^ Hz compared to PIDA HS, SCA and TLBO based which has around 7 × 10^− 4^ Hz, 7 × 10^− 4^ Hz and 7 × 10^− 4^ Hz respectively in Area (2) And also, AMPC controller has the lowest oscillations compared to PIDA HS, SCA and TLBO based in Area 1&2.

#### Robustness of AMPC against MPA-cascaded PIDA controller

**Fig. 21:**
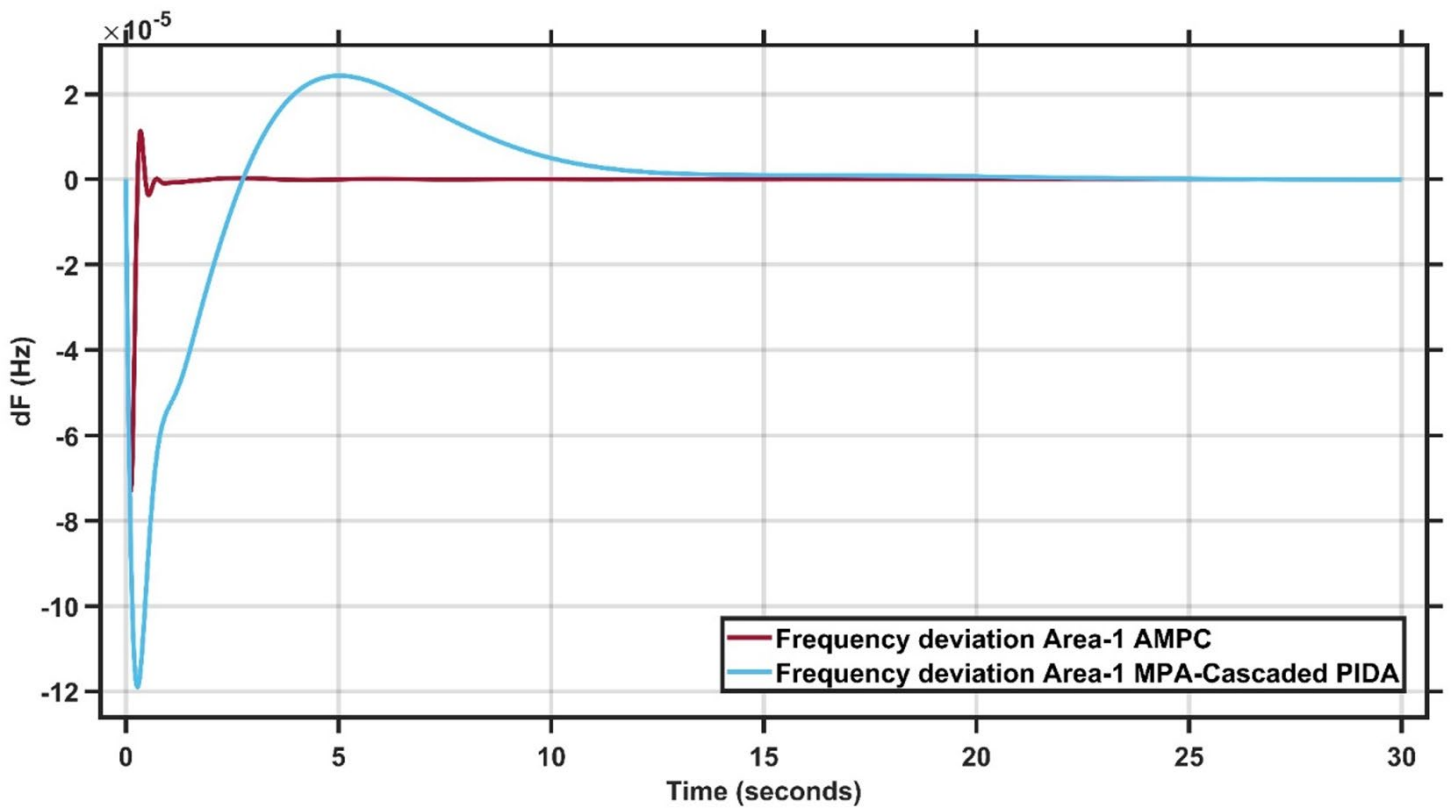
Frequency output of area 1 at 1% load disturbance in area 1&2.

**Fig. 22:**
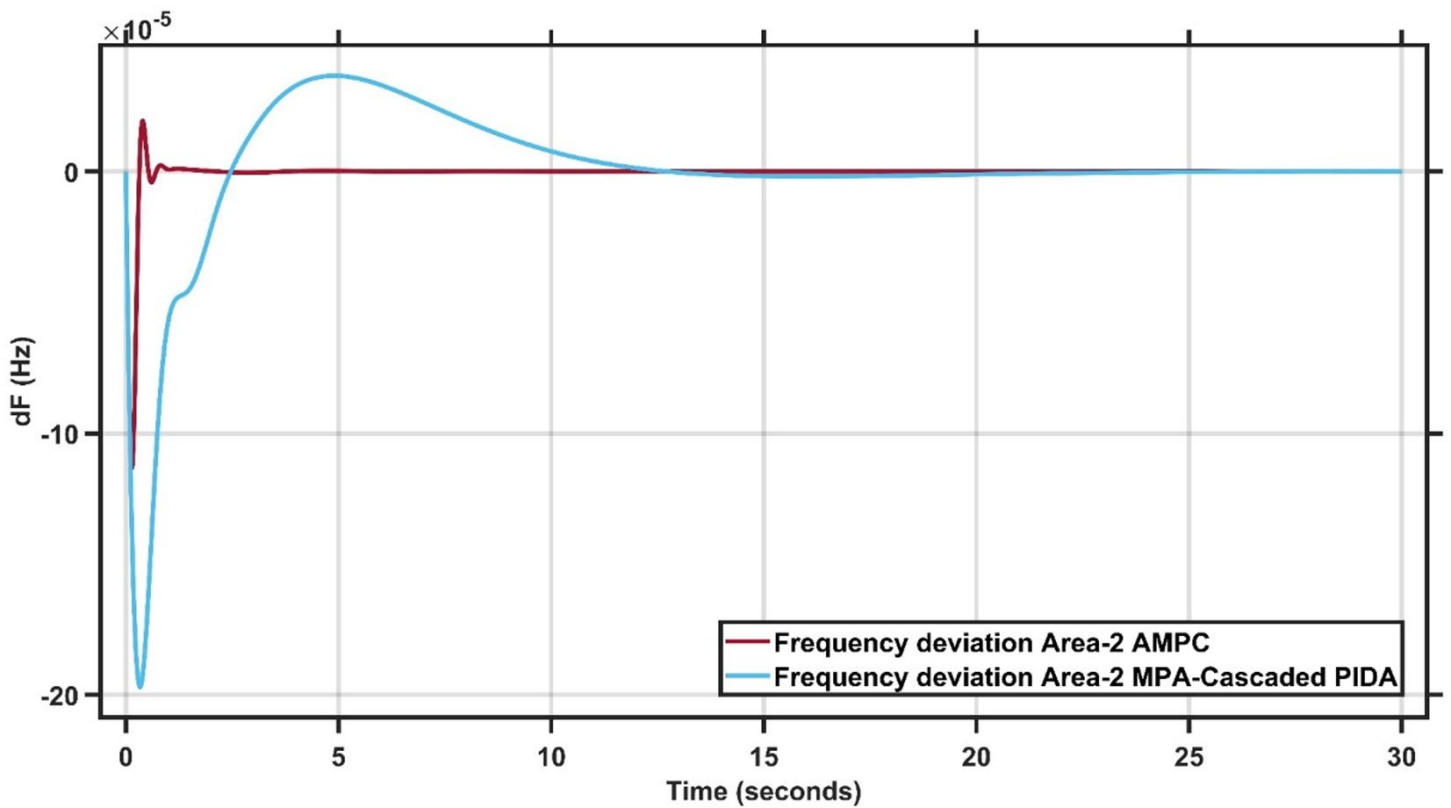
Frequency output of area 2 at 1% load disturbance in area 1&2.

In this section, 1% load disturbance is applied to Area 1 & Area 2. Figure 21 clearly demonstrates the superior performance of the AMPC controller, as the frequency output remains nearly steady throughout the simulation, whereas the “Marine Predator Algorithm” MPA-Cascaded PIDA controller exhibits noticeable oscillations and struggles to reach steady state^30^. Similarly, Fig. 22 reinforces this finding, showing that the AMPC controller provides a more stable and responsive frequency behavior compared to the MPA-Cascaded PIDA controller. Overall, Figs. 21 and 22 confirm the effectiveness of the proposed AMPC approach, which achieves steady state significantly faster and with minimal oscillations, ensuring a more stable and reliable frequency response than the MPA-Cascaded PIDA controller.

It is clear that the proposed AMPC controller has the faster settling time around 1 s compared to Cascaded-PIDA MPA based which has around 25 s in Area (1) And also, AMPC controller has the faster settling time around 1 s compared to Cascaded-PIDA MPA based which has around 25 s in Area (2) Regarding overshoot AMPC controller has the lowest overshoot around 1 × 10^− 5^ Hz compared to Cascaded-PIDA MPA based which has around 2.5 × 10^− 5^ Hz in Area (1) And also AMPC controller has the lowest overshoot around 2 × 10^− 5^ Hz compared to Cascaded-PIDA MPA based which has around 5 × 10^− 5^ Hz in Area (2) Regarding undershoot AMPC controller has the lowest undershoot around 7 × 10^− 5^ Hz compared to Cascaded-PIDA MPA based which has around 12 × 10^− 5^ Hz in Area (1) And also, AMPC controller has the lowest undershoot around 12 × 10^− 5^ Hz compared to Cascaded-PIDA MPA based which has around 20 × 10^− 5^ Hz in Area (2) And also, AMPC controller has the lowest oscillations compared to Cascaded-PIDA MPA based in Area 1&2.

### Sensitivity analysis of AMPC

**Fig. 23:**
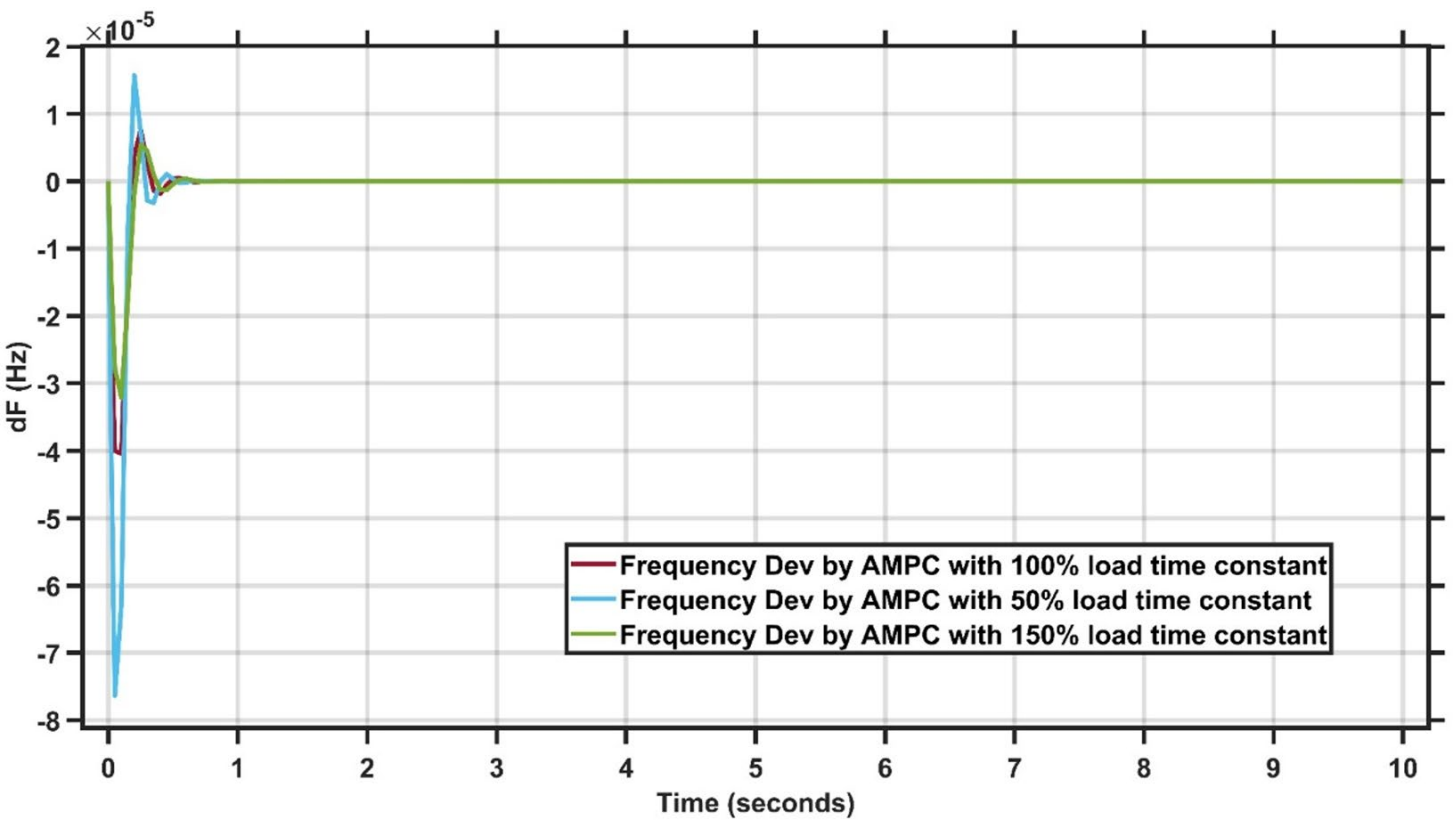
Frequency output under three conditions.

To assess the robustness of the proposed AMPC under model uncertainty, a sensitivity analysis was conducted for the single-area system under a 1% load disturbance. The load time constant was varied by − 50%, 0%, and + 50% of its nominal value, and the corresponding frequency deviations are shown in Fig. 23.

The sensitivity analysis performed on the single-area LFC system under a 1% load disturbance highlights the resilience of the AMPC framework. Even when the load time constant is varied by ± 50%, the controller preserves closed-loop stability and achieves fast frequency regulation. This robustness stems from the adaptive identification mechanism and predictive control structure, which enable the AMPC to react effectively to parameter uncertainty. Such behavior is essential for practical power systems, where load dynamics are often uncertain or time-varying.

## Conclusion

The presented simulation results verify that the proposed AMPC framework provides substantial improvements in LFC performance over conventional PI/PID and optimized PID/PIDA controllers. For single-area systems, the AMPC achieves nearly zero overshoot, reduces undershoot from 4.5 × 10⁻³ to 1 × 10⁻³, and lowers settling time from 20 to 30 s to 0–1 s, even under wind disturbances where PI exhibits peak-to-peak deviations of 26.5 × 10⁻³. For double-area systems, the AMPC restores frequency and tie-line power significantly faster, achieving 1–2 s settling compared with 20–40 s for PID, while reducing undershoot from 11.2 × 10⁻⁴ to 1.2 × 10⁻⁴ across all cases. Under dynamic disturbances, AMPC limits peak-to-peak deviations to ≤ 5 × 10⁻³, whereas PID responses reach 1.5 × 10⁻². Furthermore, when compared with HS, SCA, TLBO, and MPA-optimized controllers, the AMPC consistently yields the fastest damping, lowest oscillations, and strongest robustness. These findings confirm that the AMPC approach provides a high-performance, adaptive, and reliable control solution suited for modern, renewable-integrated power systems. In future work more complex simulations, the frequency domain analysis as the root locus and bode diagram, system uncertainty and communication time delay will be considered.

## Data Availability

The datasets used and/or analyzed during the current study are available from the corresponding author on reasonable request.
